# Lithium carbonate revitalizes tumor-reactive CD8^+^ T cells by shunting lactic acid into mitochondria

**DOI:** 10.1038/s41590-023-01738-0

**Published:** 2024-01-23

**Authors:** Jingwei Ma, Liang Tang, Yaoyao Tan, Jingxuan Xiao, Keke Wei, Xin Zhang, Yuan Ma, Shuai Tong, Jie Chen, Nannan Zhou, Li Yang, Zhang Lei, Yonggang Li, Jiadi Lv, Junwei Liu, Huafeng Zhang, Ke Tang, Yi Zhang, Bo Huang

**Affiliations:** 1 Department of Immunology, Tongji Medical College, Huazhong University of Science and Technology, Wuhan, China; 2 Department of Biochemistry & Molecular Biology, Tongji Medical College, Huazhong University of Science and Technology, Wuhan, China; 3 Department of Immunology & National Key Laboratory of Medical Molecular Biology, Institute of Basic Medical Sciences, Chinese Academy of Medical Sciences and Peking Union Medical College, Beijing, China; 4 Biotherapy Center and Cancer Center, The First Affiliated Hospital of Zhengzhou University, Zhengzhou, China; 5 Department of Oncology, The Central Hospital of Wuhan, Tongji Medical College, Huazhong University of Science and Technology, Wuhan, China; 6 Hubei Provincial Key Laboratory for Applied Toxicology, Hubei Provincial Center for Disease Control and Prevention, Wuhan, China; 7 Cardiovascular Surgery, Union Hospital, Huazhong University of Science and Technology, Wuhan, China; 8 Department of Pathology, School of Basic Medicine, Tongji Medical College, Huazhong University of Science and Technology, Wuhan, China

**Keywords:** Tumour immunology, Cancer immunotherapy, CD8-positive T cells, Biochemistry

## Abstract

The steady flow of lactic acid (LA) from tumor cells to the extracellular space via the monocarboxylate transporter symport system suppresses antitumor T cell immunity. However, LA is a natural energy metabolite that can be oxidized in the mitochondria and could potentially stimulate T cells. Here we show that the lactate-lowering mood stabilizer lithium carbonate (LC) can inhibit LA-mediated CD8^+^ T cell immunosuppression. Cytoplasmic LA increased the pumping of protons into lysosomes. LC interfered with vacuolar ATPase to block lysosomal acidification and rescue lysosomal diacylglycerol–PKCθ signaling to facilitate monocarboxylate transporter 1 localization to mitochondrial membranes, thus transporting LA into the mitochondria as an energy source for CD8^+^ T cells. These findings indicate that targeting LA metabolism using LC could support cancer immunotherapy.

## Abstract (web summary)

Here the authors show that lithium carbonate can revitalize tumor-reactive CD8^+^T cells by shunting cytosolic lactic acid into the mitochondria for oxidation, indicating that lithium ions might be applied as a cancer immunotherapy.

## Main

Human tumor cells produce up to 40 times more LA than normal cells, and up to 40 μmol per gram of LA is present in tumor microenvironments^1,2^. Thus, tumor-infiltrating lymphocytes (TILs) are considered to be soaked in LA-enriched fluid, where LA is transported into the cytoplasm of TILs and dampens the cytolysis of tumor cells through largely unknown mechanisms^3–5^. However, a higher pH (less H^+^) favors glycolytic enzymes to exert their activities^6^, which may partially explain the LA-mediated suppression. Clinically, high LA levels are correlated with poor prognosis in cancer patients^7–9^. These findings together suggest that LA is a potential immune checkpoint molecule in tumor microenvironments^10,11^. Nevertheless, LA per se is a type of energy source that can be physiologically utilized through the Cori cycle or directly oxidized in the mitochondria^12,13^. Whether and how LA can be used as a carbon source to revitalize tumor-reactive CD8^+^ T cells is unclear.

## Results

### LC channels exogenous LA into mitochondria for oxidation

**Fig. 1:**
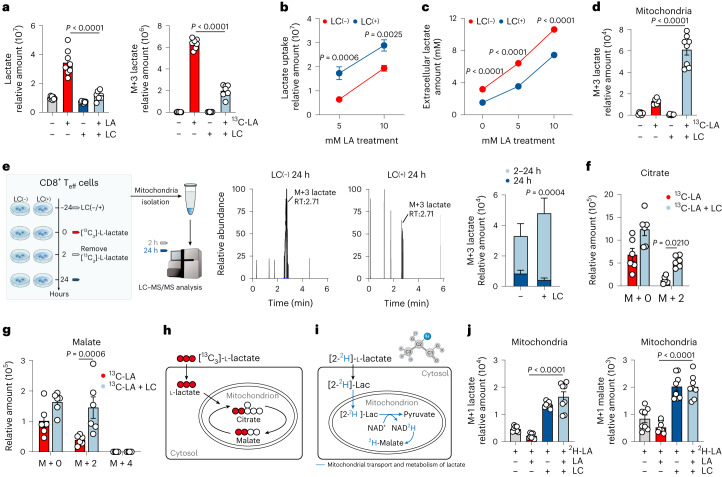
LC channels exogenous LA into mitochondria for oxidation. **a**, Relative abundance, determined by LC–MS/MS, of intracellular lactate (left) or M + 3 lactate (right) in C57BL/6J mice spleen-derived and CD3/CD28 beads activated CD8^+^ T_eff_ cells treated with 10 mM unlabeled LA (pH 6.6) or ^13^C-labeled LA (pH 6.6) and/or LC for 24 h. *n* = 8. **b**, Uptake of lactate by CD8^+^ T_eff_ cells treated with LA (5/10 mM) and/or LC, determined at 0/30 min by LC–MS/MS. Relative uptake amount = relative amount_30min _− relative amount_0min_. *n* = 10. **c**, Extracellular lactate concentration in culture medium derived from CD8^+^ T_eff_ cells treated with 10 mM LA and/or LC. *n* = 5. **d**, Relative abundance, determined by LC–MS/MS, of mitochondrial M + 3 lactate in CD8^+^ T_eff_ cells pretreated with LC for 24 h then treated with or without 10 mM ^13^C-labeled lactate for 2 h. *n* = 8 in LC-treated groups and *n* = 6 in non-LC groups. **e**, As in **d**, then removed ^13^C-labeled lactate for another 22-h culture, determined at 2/24 h. Lactate consumption amount = relative amount_2h _− relative amount_24h_. *n* = 9. **f**,**g**, Relative abundance, determined by LC–MS/MS, of intracellular citrate (**f**) and malate (**g**) derived from ^13^C-labeled lactate as in **a** (right). *n* = 6. **h**, Schematic showing incorporation of ^13^C derived from lactate into downstream metabolites. **i**, Schematic showing mitochondrial transport and metabolism of deuterated lactate (2-^2^H-Lac). Created with BioRender.com. **j**, Relative abundance, determined by LC–MS/MS, of mitochondrial M + 1 lactate and M + 1 malate in CD8^+^ T_eff_ cells treated with LA and/or LC and 2 mM ^2^H-LA for 2 h. *n* = 8. Data are the mean ± s.e.m. *n* = biological replicates unless stated otherwise. *P* values were calculated using unpaired two-tailed Student’s *t*-test (**e**–**g**), one-way analysis of variance (ANOVA) for Dunnett’s multiple-comparisons test (**a**, **d** and **j**) and two-way ANOVA for Sidak’s multiple-comparisons test (**b** and **c**). Source data

**Extended Data Fig. 1:**
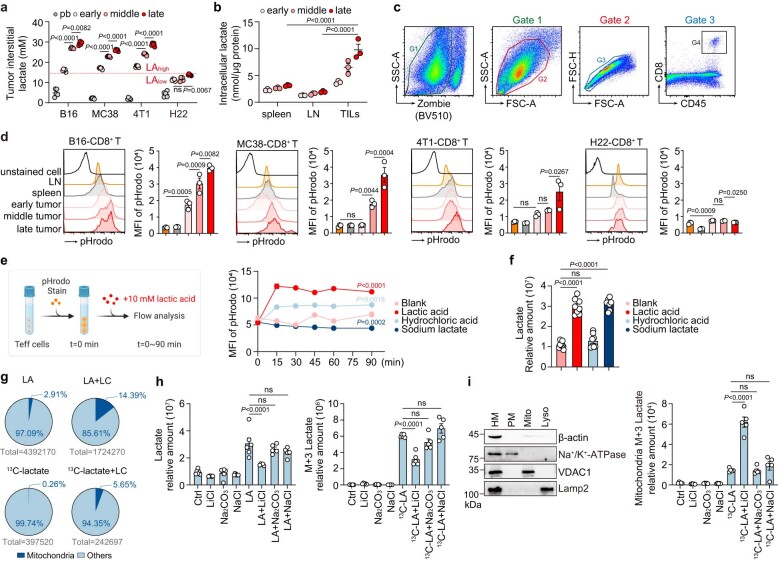
LC channels exogenous LA into mitochondria for oxidation. **a**, Lactate concentration in tumor interstitial fluids of B16/MC38/4T1 tumor-bearing mice and H22 ascites mice at different stages of tumor/ascites growth (tumor size/ascites volume: early 0-500 mm^3^/0-1 mL; middle 500–1000 mm^3^/1–3 mL; late 1000–2000 mm^3^/3–6 mL), above the dotted line represents LA_high_, and below represents LA_low_. Peripheral blood (pb) was used as control. *n* = 5. **b**, Intracellular lactate concentration of CD8^+^ T cells in spleen, LN and TILs of B16 tumor-bearing mice at different stages of tumor growth as in **a**. *n* = 3. **c**, Flow cytometry gate strategy of Zombie^-^CD45^+^CD8^+^ T cells in tumor-bearing mice. **d**, Cytosolic acidification, detected by flow cytometry, of CD45^+^CD8^+^ T cells as in **c** from LN, spleen and tumor in B16/MC38/4T1 tumor-bearing mice and H22 ascites mice. *n* = 3. **e**, Diagram was created by BioRender.com (left). Cytosolic acidification of C57BL/6J mice spleen-derived and CD3/CD28 beads activated CD8^+^ Teff cells treated with 10 mM lactic acid, hydrochloric acid or sodium lactate (right). *n* = 3. **f**, Relative abundance, determined by LC-MS/MS, of intracellular lactate in CD8^+^ Teff cells as in **e**. *n* = 8. **g**, Relative abundance of intracellular lactate or M+3 lactate in CD8^+^ Teff cells treated with 10 mM unlabelled LA/^13^C-labelled lactate and/or LC for 24 hrs. **h**, Relative abundance of intracellular lactate (left) or M+3 lactate (right) in CD8^+^ Teff cells treated with 10 mM unlabelled LA (pH 6.6) or ^13^C-LA (pH 6.6) and/or LiCl/Na_2_CO_3_/NaCl for 24 hrs. *n* = 6 in Ctrl/LiCl/ Na_2_CO_3_/NaCl/LA group, *n* = 5 in other groups. **i**, Western blotting against VDAC1 (mitochondrial membrane, Mito), Na^+^/K^+^-ATPase (plasma membrane, PM), Lamp2 (lysosomal membrane, Lyso) and β-actin (homogenate, HM) (left). Relative abundance of mitochondrial M+3 lactate in CD8^+^ Teff cells pretreated with LiCl/Na_2_CO_3_/NaCl then treated with or without 10 mM ^13^C-lactate for 2 hrs (right). *n* = 5. Data are mean±s.e.m. *n* = biological replicates unless stated otherwise. *P* values were calculated using one-way ANOVA for Dunnett’s multiple comparisons test (a, d, f, h and i), two-way ANOVA for Tukey’s multiple comparisons test (b, e), ns (not significant). Source data

**Extended Data Fig. 2:**
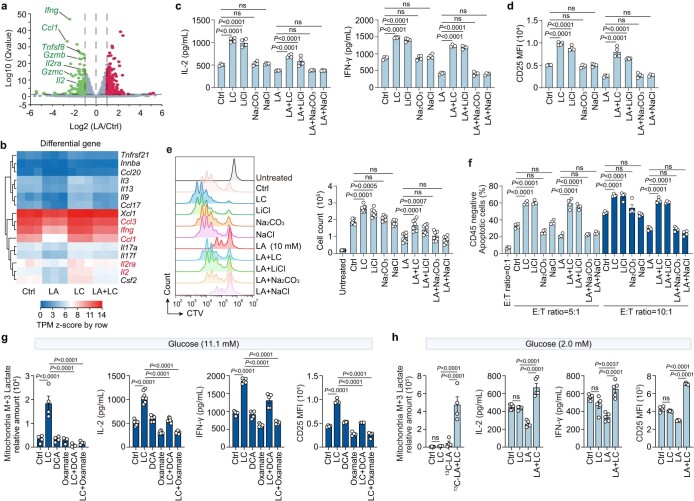
LC reverses the LA-suppressive effects in CD8+ Teff cells. **a**, Volcano plot of differential expression of CD8^+^ Teff cells treated with or without 10 mM LA for 3 hrs. CD8^+^ Teff cell activation relative genes were annotated. *n* = 3. **b**, Signature genes expressed by CD8^+^ Teff cells treated with LA and/or LC, identified through RNA-sequencing (RNA-seq) analysis. *n* = 3. **c**, IL-2 and IFN-γ in supernatant, detected by ELISA, of CD8^+^ Teff cells treated with 10 mM LA and/or LC/LiCl/Na_2_CO_3_/NaCl for 24 hrs. *n* = 4. **d**, CD25 expression (MFI), analyzed by flow cytometry, of CD8^+^ Teff cells treated as in **c**. *n* = 4. **e**, Proliferation, analyzed by flow cytometry, and cell counts of CTV-labeled CD8^+^ T cells treated with 10 mM LA and/or LC/LiCl/Na_2_CO_3_/NaCl for 72 hrs. *n* = 8. **f**, OVA-peptide primed OT-1 mice derived CD8^+^ Teff cells were treated with 10 mM LA plus LC, LiCl, Na_2_CO_3_ or NaCl for 24 hrs, and incubated with OVA-B16 tumor cells for 8 hrs. CD45^-^ cell apoptosis was determined by flow cytometry. *n* = 4. **g**, Relative abundance of mitochondrial M+3 lactate in CD8^+^ Teff cells pretreated with LC and/or DCA or Oxamate under the 11.1 mM ^13^C-glucose culture (*n* = 4). IL-2 (*n* = 7) and IFN-γ (*n* = 5) in supernatant, CD25 expression (*n* = 4), of CD8^+^ Teff cells treated with LC and/or DCA/Oxamate under the 11.1 mM glucose culture. **h**, Relative abundance of mitochondrial M+3 lactate in CD8^+^ Teff cells pretreated with LC and treated with or without 10 mM ^13^C-lactate under the 2 mM ^13^C-glucose culture (*n* = 4). IL-2 (*n* = 5) and IFN-γ (*n* = 5) in supernatant, CD25 expression (*n* = 5), of CD8^+^ Teff cells treated with LC and/or LA under the 2 mM glucose culture. Data are mean±s.e.m. *n* = biological replicates unless stated otherwise. *P* values were calculated using one-way ANOVA for Dunnett’s multiple comparisons test (c, d, f, g and h), ns (not significant). Source data

Tissue LA exists in the form of lactate and hydrogen ions (H^+^ or protons) that are symported into the cytosol of cells via monocarboxylate transporter 1 (MCT1)^14^. In parallel with gradually elevated extracellular lactate levels in B16, MC38 and 4T1 tumor tissues (LA_high_ tumor; Extended Data Fig. 1a), intracellular lactate ions in tumor-infiltrating CD8^+^ T cells correspondingly increased (Extended Data Fig. 1b), concomitant with the cytosolic acidification (Extended Data Fig. 1c,d). However, these results were not observed in the H22 hepatocarcinoma ascites model (LA_low_ tumor; Extended Data Fig. 1a,d). In addition, neither hydrochloric acid nor sodium lactate, when used to treat activated CD8^+^ T_eff_ cells, rapidly and simultaneously increased the intracellular H^+^ and lactate levels (Extended Data Fig. 1e,f). ^13^C-labeled lactate tracing showed negligible lactate in the mitochondria of LA-treated CD8^+^ T_eff_ cells (Extended Data Fig. 1g) despite that LA can be oxidized in the mitochondria^15^. To identify an agent that can allot exogenous LA to mitochondria for oxidation, we explored LC, because its monotherapy has been shown to lower lactate levels in patients with bipolar depression^16^. Intracellular lactate levels were increased in the CD8^+^ effector T (T_eff_) cells by LA, which could be counteracted by 0.6 mM LC or 1.2 mM lithium chloride (LiCl) treatment, but not by sodium carbonate (Na_2_CO_3_) or sodium chloride (NaCl) treatment (Fig. 1a and Extended Data Fig. 1h). Similar results were also obtained from ^13^C-labeled lactate tracing (Fig. 1a and Extended Data Fig. 1h). This observation might be explained by the fact that LC (1) inhibited the uptake of extracellular LA, (2) promoted the excretion of intracellular LA, or (3) remodeled intracellular LA metabolism. We found that LC did not inhibit the uptake of exogenous LA (Fig. 1b); in contrast, LC decreased extracellular lactate (Fig. 1c), suggesting that LC redistributes LA. ^13^C-labeled lactate tracing showed that M + 3 lactate was highly accumulated in the mitochondria of CD8^+^ T_eff_ cells treated with LC or LiCl, but not with Na_2_CO_3_ or NaCl (Fig. 1d and Extended Data Fig. 1i). After removing ^13^C-labeled lactate at 2 h, M + 3 lactate was rarely detected 24 h later (Fig. 1e). In line with these results, the relative abundance of tricarboxylic acid (TCA) cycle intermediates, including citrate and malate, was markedly elevated in response to LC treatment (Fig. 1f–h). Here, we additionally used [2-^2^H]lactate to trace lactate and found that M + 1 lactate and M + 1 malate were present in the mitochondria (Fig. 1i,j), suggesting that lactate is not initially converted to pyruvate in the cytosol but directly imported into the mitochondria where lactate can be oxidized to supply energy for CD8^+^ T_eff_ cells. Using unlabeled LA or ^13^C-labeled lactate to further trace intracellular lactate distribution, we found that LC treatment increased unlabeled lactate from 2.91% to 14.39% and labeled M + 3 lactate from 0.26% to 5.65% in the mitochondria (Extended Data Fig. 1g). In line with this, we found that only LC and LiCl, but not Na_2_CO_3_ or NaCl, reversed the suppressive effects of LA on the activation, proliferation and cytolysis of CD8^+^ T_eff_ cells in vitro (Extended Data Fig. 2a–f). Moreover, when we used high [^13^C]glucose (11.1 mM) and low [^13^C]glucose (2 mM) to culture T_eff_ cells, we found that LC treatment promoted M + 3 lactate accumulation in the mitochondria of high-glucose-cultured T_eff_ cells, which was counteracted by sodium dichloroacetate or sodium oxamate; in contrast, M + 3 lactate was not accumulated in mitochondria of low-glucose-cultured T_eff_ cells, which was rescued by the addition of exogenous LA (Extended Data Fig. 2g,h), suggesting that LC exerts its promoting effect on CD8^+^ T cells in an LA-dependent way.

### LC-activated PKCθ facilitates MCT1 entry to mitochondria

**Fig. 2:**
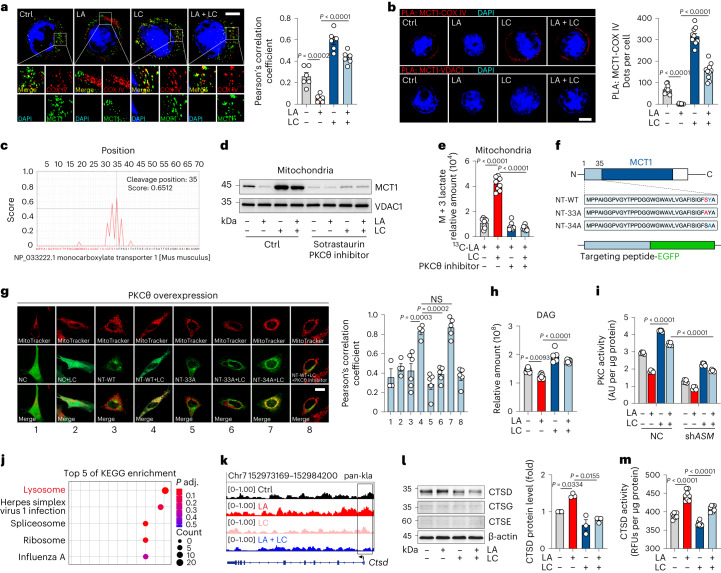
LC facilitates MCT1 entry into mitochondria. **a**, Immunofluorescence staining of MCT1 and COX IV in CD8^+^ T_eff_ cells treated with LA and/or LC. Nuclei were stained with DAPI. Scale bar, 5 μm (left). Pearson’s correlation coefficient between MCT1 and COX IV (right). *n* = 6 cells examined over three independent experiments. **b**, PLA showing the interaction between COX IV (but not the VDAC1) and MCT1 in CD8^+^T_eff_ cells treated as in **a**. Scale bar, 5 μm. Quantification of PLA-positive foci per cell (right). *n* = 10 cells examined over three independent experiments. **c**, PrediSi prediction for the mitochondria-targeting peptide sequence of MCT1. **d**, Immunoblots of MCT1 in the mitochondria of CD8^+^ T_eff_ cells treated with LA and/or LC/sotrastaurin. **e**, Relative abundance of mitochondrial M + 3 lactate in CD8^+^ T_eff_ cells treated with ^13^C-labeled LA and/or LC/sotrastaurin. *n* = 8. **f**, Schematic sequence of the N terminus (NT) of MCT1 and diagram of targeting peptide–EGFP fusion protein. **g**, Colocalization of targeting peptide fragments fused to EGFP with MitoTracker in HeLa cells. Scale bar, 20 μm (left). Pearson’s correlation coefficient between EGFP and MitoTracker (right). *n* = 3 in group 1, *n* = 4 in group 2/4, *n* = 5 in group 5/6/7/8, *n* = 6 in group 3. **h**, Relative abundance of intracellular DAG in CD8^+^ T_eff_ cells treated with LA and/or LC. *n* = 6. **i**, PKC activity of CD8^+^ T_eff_ cells transduced with sh*ASM* and treated with LA and/or LC. AU, absorbance unit. *n* = 5. **j**, ChIP–seq analysis was performed with an antibody to pan-Kla in CD8^+^ T_eff_ cells. KEGG analysis showed the top five differential enrichment pathways with lactylation. **k**, Representation of ChIP–seq and input profiles at the *Ctsd* gene locus. **l**, Immunoblots of CTSD, CTSG and CTSE in CD8^+^ T_eff_ cells (left). Quantification of CTSD expression from immunoblotting (right). The control was set to 1. *n* = 3. **m**, CTSD activity was detected in CD8^+^ T_eff_ cells. RFUs, relative fluorescence units. *n* = 8. Data are the mean ± s.e.m. *n* = biological replicates unless stated otherwise. *P* values were calculated using one-way ANOVA for Dunnett’s multiple-comparisons test (**a**, **b**, **g**, **h**, **l** and **m**) and two-way ANOVA for Tukey’s multiple-comparisons test (**e** and **i**); NS, not significant. Source data

**Extended Data Fig. 3:**
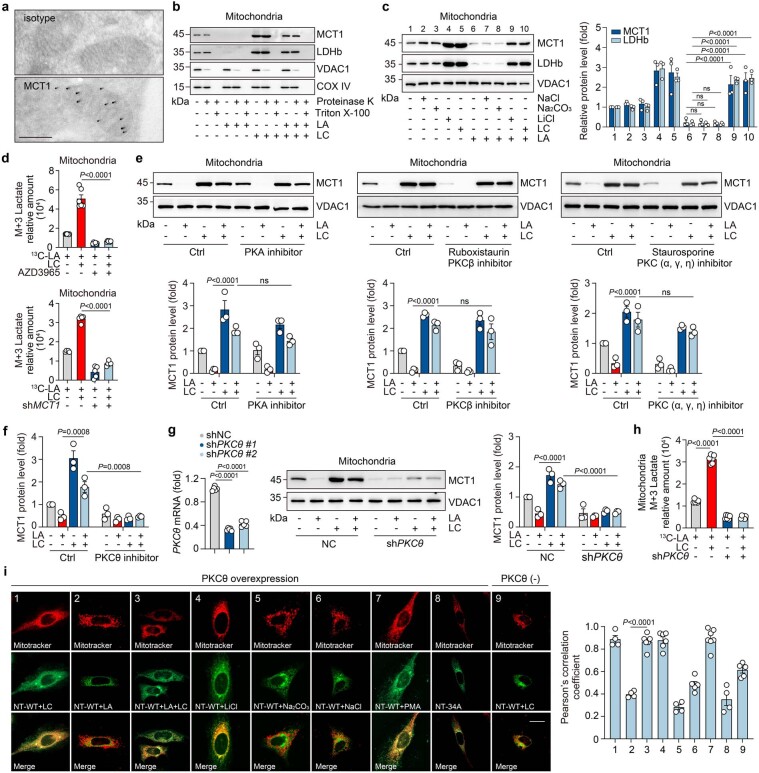
LC-activated PKCθ facilitates MCT1 entry into mitochondria. **a**, Electron micrographs of cryosectioned CD8^+^ Teff cells immunolabeled with anti-MCT1 and secondary antibody conjugated to 10 nm colloidal gold. Scale bar, 200 nm. *n* = 3 independent experiments. **b**, Immunoblots of mitochondria compartment isolated from CD8^+^ Teff cells treated with LA and/or LC then treated with proteinase K (100 ng/mL) in the absence or presence of 1% Triton X-100. **c**, Immunoblots of MCT1/LDHb in the mitochondria of CD8^+^ Teff cells (left). Quantification of MCT1/LDHb expression from western blotting (right). The control was set to 1. *n* = 3. **d**, Relative abundance of mitochondrial M+3 lactate in CD8^+^ Teff or sh*MCT1*/sh*NC* CD8^+^ Teff cells pretreated with LC and/or 500 μM AZD3965 then treated with ^13^C-lactate for 2 hrs. *n* = 6/5 in top/bottom groups. **e**, Immunoblots of MCT1 in the mitochondria of CD8^+^ Teff cells treated with LA and/or LC/PKA inhibitor/ruboxistaurin/staurosporine (top). Quantification of MCT1 expression from western blotting (bottom). The control was set to 1. *n* = 3. **f**, Quantification of MCT1 expression from western blotting in Fig. 2d. *n* = 3. **g**, Silencing efficiency detection of sh*PKCθ* in CD8^+^ Teff cells (left). *n* = 5. Immunoblots of MCT1 in the mitochondria of CD8^+^ Teff cells transduced with sh*PKCθ* and then treated with LA and/or LC (middle). Quantification of MCT1 expression from western blotting (right). The control was set to 1. *n* = 3. **h**, Relative abundance of mitochondrial M+3 lactate in CD8^+^ Teff cells transduced with sh*PKC*/sh*NC* and treated with or without LC for 24 hrs and treated with ^13^C-lactate for 2 hrs. *n* = 6. **i**, Colocalization of targeting peptide fragments fused to EGFP with Mito-tracker in Hela cells transfected with targeting peptide-EGFP and/or *PKCθ*-overexpression plasmids. Scale bar, 20 μm (left). Pearson’s correlation coefficient between EGFP and Mito-tracker (right). *n* = 4 in group 1/2/5/8, *n* = 5 in group 6/9, *n* = 6 in group 3/4, *n* = 7 in group 7. Data are mean±s.e.m. *n* = biological replicates unless stated otherwise. *P* values were calculated using one-way ANOVA for Dunnett’s multiple comparisons test (c, g left and i), two-way ANOVA for Tukey’s multiple comparisons test (e, f and g right), ns (not significant). Source data

Next, we investigated the molecular basis by which LC guides the distribution of cytosolic LA into mitochondria. The translocation of LA to the mitochondria requires MCT1 to localize to the inner mitochondrial membrane (IMM)^8^. In our study, we found that LC treatment resulted in the localization of MCT1 to the IMM of CD8^+^ T_eff_ cells, as evidenced by super-resolution fluorescence microscopy and transmission electron microscopy with the immunogold labeling method (Fig. 2a and Extended Data Fig. 3a), consistent with the previous proposal of mitochondrial lactate oxidation complex, composed of MCT1, LDHb, CD147 and cytochrome oxidase IV (COX IV)^17^. Using a proximity ligation assay (PLA), we found that MCT1 had strong PLA signals with COX IV, but not VDAC1 (OMM protein) after LC treatment (Fig. 2b). In addition, we digested mitochondrial fractions of CD8^+^ T_eff_ cells with proteinase K. Immunoblot analysis showed that MCT1 was expressed in the IMM of LC-treated fractions (Extended Data Fig. 3b). Similar results were also obtained from LiCl-treated CD8^+^ T_eff_ cells. However, neither Na_2_CO_3_ nor NaCl induced mitochondrial MCT1 expression in CD8^+^ T_eff_ cells (Extended Data Fig. 3c). In line with these results, use of the MCT1 inhibitor AZD3965 or sh*MCT1* abrogated ^13^C-labeled lactate mitochondrial accumulation in LC-treated CD8^+^ T_eff_ cells (Extended Data Fig. 3d). MCT1 is a secretory protein that has an endoplasmic reticulum (ER)-targeting sequence that allows MCT1 to enter the ER and localize to the plasma membrane^14^. To reconcile this paradoxical mitochondrial location, we speculated that LC might induce an MCT1 variant with a mitochondria-targeting sequence (MTS). However, sequencing analysis did not find MCT1 variants in LC-treated CD8^+^ T_eff_ cells (Supplementary Table 1). Notwithstanding this, we found that the ER-targeting sequence of MCT1 at the N terminus was followed by MTS (Fig. 2c). Such a bimodal sequence might guide MCT1 to target either the ER or the mitochondria, which may be regulated by serine/threonine phosphorylation by protein kinase A (PKA) or protein kinase C (PKC)^18^. Using PKA, PKCβ or PKC (α, γ, η) inhibitors did not significantly alter the LC-induced MCT1 location in IMM (Extended Data Fig. 3e); however, the MCT1 location in the IMM and LA entry was almost completely blocked by the PKCθ inhibitor sotrastaurin (Fig. 2d,e and Extended Data Fig. 3f). Knockdown of *Prkcq* with shRNA also prevented the location of MCT1 in the IMM and LA entry (Extended Data Fig. 3g,h). To verify the interaction of PKCθ with the MTS of MCT1, the N-terminal fragment of MCT1 with or without a point mutation was fused to the enhanced green fluorescent protein (EGFP; Fig. 2f). Co-transfection of PKCθ and targeting peptide–EGFP (NT-WT) or mutated peptide–EGFP (NT-33A or NT-34A) into HeLa cells, we found that despite the weak mitochondrial localization signal of EGFP in the NT-WT group, LC treatment forced EGFP localization to the mitochondria; however, the NT-33A mutation rather than NT-34A disrupted the mitochondrial localization of EGFP (Fig. 2g). In addition, the use of a PKCθ inhibitor or transfection of peptide–EGFP alone did not result in MCT1 localization to the mitochondria (Fig. 2g and Extended Data Fig. 3i). Consistently, neither Na_2_CO_3_ nor NaCl induced colocalization of EGFP with mitochondria in CD8^+^T_eff_ cells (Extended Data Fig. 3i). These results suggest that LC facilitates the localization of MCT1 to the mitochondria by promoting PKCθ-mediated phosphorylation of MCT1-MTS.

### PKCθ activation is induced by LC-regulated lysosomal ASM

**Extended Data Fig. 4:**
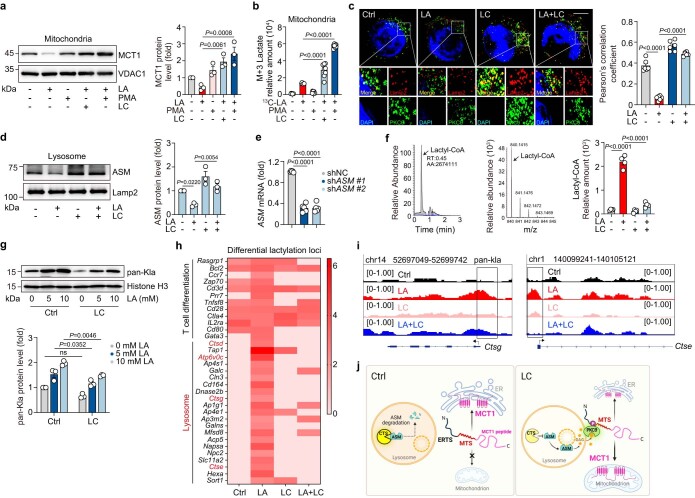
LC facilitates MCT1 entry into mitochondria in a lysosomal cathepsin D-ASM-DAG-PKCθ-dependent manner. **a**, Immunoblots of MCT1 in the mitochondria of CD8^+^ Teff cells treated with LA and/or LC in the presence or absence of PMA for 48 hrs (left). Quantification of MCT1 expression from western blotting (right). The control was set to 1. *n* = 3. **b**, Relative abundance, determined by LC-MS/MS, of mitochondrial M+3 lactate in CD8^+^ Teff cells treated with or without LC in the presence or absence of PMA for 24 hrs and then treated with ^13^C-lactate for 2 hrs. *n* = 8. **c**, Immunofluorescence staining of PKCθ (green) and Lamp2 (red) in CD8^+^ Teff cells. Scale bar, 5 μm (left). Pearson’s correlation coefficient between PKCθ and Lamp2 (right). *n* = 6 cells examined over 3 independent experiments. **d**, Immunoblots of ASM in the lysosome of CD8^+^ Teff cells treated with LA and/or LC for 48 hrs (left). Quantification of ASM expression from western blotting (right). The control was set to 1. *n* = 3. **e**, Silencing efficiency detection of sh*ASM* in CD8^+^ Teff cells. *n* = 5. **f**, LC-MS/MS was performed for the L-lactyl-CoA standard (left and middle). Relative abundance, determined by LC-MS/MS, of intracellular lactyl-CoA in CD8^+^ T cells treated with LA and/or LC (right) for 24 hrs. *n* = 4. **g**, Immunoblots of pan-Kla in CD8^+^ Teff cells treated with LA and/or LC (top). Quantification of pan-Kla expression from western blotting (bottom). The control was set to 1. *n* = 3. **h**, Heatmap displayed differential lactylation loci in CD8^+^ T cells treated with LA and/or LC. **i**, Representation of ChIP-seq and input profiles at the *Ctsg* and *Ctse* gene locus. **j**, Proposed model for LC-induced PKCθ activation and MCT1 translocation to mitochondria. Created with BioRender.com. Data are mean±s.e.m. *n* = biological replicates unless stated otherwise. *P* values were calculated using one-way ANOVA for Dunnett’s multiple comparisons test (a, b, c, d, e and f), two-way ANOVA for Tukey’s multiple comparisons test (g), ns (not significant). Source data

PKCθ belongs to a novel PKC subfamily, which is activated by diacylglycerol (DAG)^19^ and is widely expressed in T lymphocytes^20^. We found that LC treatment elevated the DAG levels in CD8^+^ T_eff_ cells (Fig. 2h) and the use of the DAG analog phorbol esters (PMA) resulted in MCT1 localization to and LA accumulation in mitochondria (Extended Data Fig. 4a,b). We then addressed the mechanism by which LC treatment triggered PKCθ activation. We found that LC treatment resulted in PKCθ approaching lysosomes (Extended Data Fig. 4c), suggesting a possibility that DAG is derived from lysosomes by lysosomal phospholipase. Although lysosomes do not seem to express conventional phospholipase C (PLC) enzymes that catalyze phospholipids to generate DAG, this organelle expresses acid sphingomyelinase (ASM), which also possesses PLC activity^21^. We found that LC treatment elevated lysosomal ASM levels (Extended Data Fig. 4d), concomitant with an increase in DAG levels. Then, we knocked down *ASM* (Extended Data Fig. 4e); under such conditions, we found that PKCθ activation could not be induced by LC treatment in the above CD8^+^ T_eff_ cells (Fig. 2i), suggesting that PKCθ is activated by lysosomal ASM-induced DAG. LA accumulation may result in histone lysine lactylation (Kla) in the cells and promote gene expression^22^. Both lactyl-CoA (a direct donor for lactylation) and pan-histone Kla were increased in CD8^+^ T_eff_ cells, as evidenced by liquid chromatography–tandem mass spectrometry (LC–MS/MS) analysis and immunoblotting, which, however, was counteracted by LC (Extended Data Fig. 4f,g). ASM has been reported to be degraded by cathepsins^23^. Intriguingly, chromatin immunoprecipitation with sequencing (ChIP–seq) analysis showed that LA-induced histone Kla was enriched in the lysosomal pathway by KEGG (Kyoto Encyclopedia of Genes and Genomes) analysis (Fig. 2j), revealing that lysosome-associated cathepsin genes (*Ctsd*, *Ctsg* and *Ctse*) were lactylated in LA-treated CD8^+^ T_eff_ cells; however, the lactylation was removed by LC treatment (Fig. 2k and Extended Data Fig. 4h,i). In addition, we found that LC treatment decreased the expression and activity of cathepsin D, but not cathepsins G and E (Fig. 2l,m). Together, these results suggest that LC treatment activates PKCθ via lysosomal ASM, thus facilitating MCT1 translocation to mitochondria (Extended Data Fig. 4j).

### LC triggers TFEB to transcriptionally activate LDHB

**Fig. 3:**
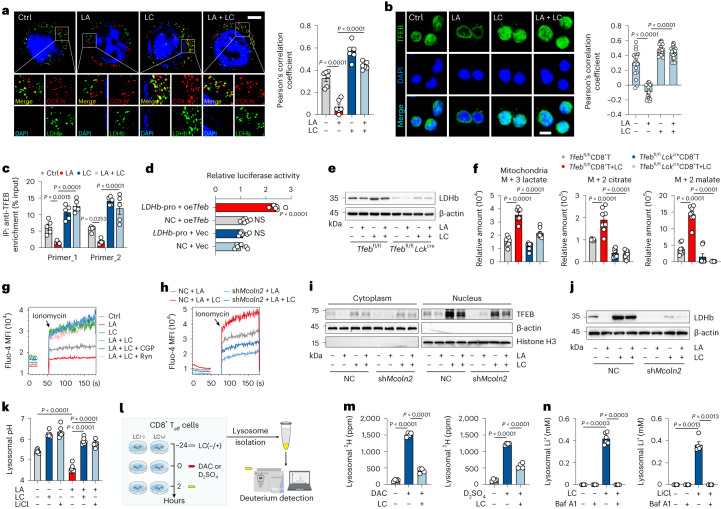
LC triggers TFEB to transcriptionally activate LDHB. **a**, Immunofluorescence staining of LDHB and COX IV in CD8^+^ T_eff_ cells treated with LA and/or LC. Scale bar, 5 μm (left). Pearson’s correlation coefficient between LDHB and COX IV (right). *n* = 6 cells examined over three independent experiments. **b**, Immunofluorescence staining of TFEB in CD8^+^ T_eff_ cells as in **a**. Scale bar, 10 μm (left). Pearson’s correlation coefficient between TFEB and DAPI (right). *n* = 20 cells examined over three independent experiments. **c**, ChIP–qPCR analysis of TFEB enrichment around the promoter of *LDHB* in CD8^+^ T_eff_ cells. *LDHB* primer 1 and 2 were used. *n* = 5. **d**, Relative luciferase activity of HEK-293T cells transfected with *Tfeb*-overexpressing (oe*Tfeb*) or *LDHB*-promoter (*LDHB*-pro) plasmids. *n* = 6. **e**, Immunoblots of LDHB in *Tfeb*^fl/fl^ or *Tfeb*^fl/fl^ *Lck*^cre^ mice spleen-derived CD8^+^ T_eff_ cells. **f**, Relative abundance of mitochondrial labeled lactate, citrate and malate derived from [^13^C]lactate in CD8^+^ T_eff_ cells of *Tfeb*^fl/fl^ or *Tfeb*^fl/fl^ *Lck*^cre^ mice. *n* = 7 in M + 2 citrate *Tfeb*^fl/fl^ CD8^+^ T group, *n* = 8 in other groups. **g**, Fluo-4 mean fluorescence intensity (MFI) of cytosolic calcium release in CD8^+^ T_eff_ cells pretreated with CGP37157 (CGP, 10 μM) or ryanodine (Ryn, 100 μM) for 1 h then treated with LA and/or LC. **h**, Fluo-4 MFI of cytosolic calcium release in CD8^+^ T_eff_ cells pre-transduced with sh*Mcoln2* then treated with LA and/or LC. **i**,**j**, Immunoblots of TFEB (**i**) and LDHB (**j**) in CD8^+^ T_eff_ cells as in **h**. **k**, Lysosomal pH value of CD8^+^ T_eff_ cells was detected. *n* = 6. **l**, Schematic of experimental design for deuterium detection. Created with BioRender.com. **m**, Lysosomal ^2^H concentration, determined by isotope-MS, of CD8^+^ T_eff_ cells with or without deuterated ^2^H-acetic acid (DAC, 10 mM) or ^2^H-sulfuric acid (D_2_SO_4_, 5 mM) for 3 h. *n* = 4. **n**, Lysosomal Li^+^ concentration in CD8^+^ T_eff_ cells treated with LC/LiCl and/or bafilomycin A1 (Baf A1, 1 nM) was detected. *n* = 6 (left) and *n* = 5 (right). Data are the mean ± s.e.m. *n* = biological replicates unless stated otherwise. *P* values were calculated using one-way ANOVA for Dunnett’s multiple-comparisons test (**a**–**d**, **f**, **k**, and **m**) and Kruskal–Wallis test (**n**). Source data

**Extended Data Fig. 5:**
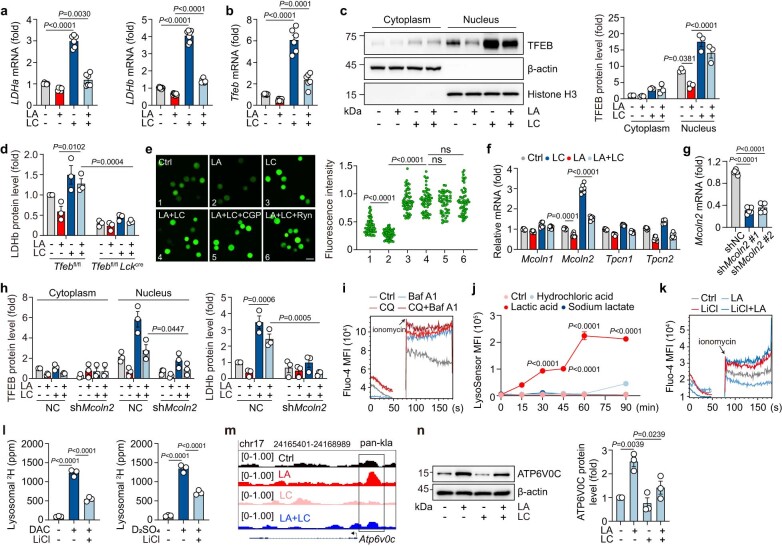
LC triggers TFEB to transcriptionally activate LDHb. **a** and **b**, *Ldha* (*n* = 6) and *Ldhb* (*n* = 7) expression (a), *Tfeb* (*n* = 6) expression (b), analyzed by PCR, of CD8^+^ Teff cells treated with LA and/or LC for 24 hrs. **c**, Immunoblots of TFEB in CD8^+^ Teff cells as in **a**. Quantification of TFEB expression in nucleus from western blotting. The control was set to 1. *n* = 3. **d**, Quantification of LDHb expression from western blotting in Fig. 3e. *n* = 3. **e**, Fluo-4 fluorescence imaging, of cytosolic calcium release in CD8^+^ Teff cells pretreated with CGP37157 or ryanodine for 1 hr then treated with LA and/or LC. Fluorescence intensity of Fluo-4 signals per cell in left. *n* = 55 cells examined over 3 independent experiments. **f**, *Mcoln1*, *Mcoln2, Tpcn1* and *Tpcn2* expression of CD8^+^ Teff cells treated with LA and/or LC for 24 hrs. *n* = 7. **g**, Silencing efficiency detection of sh*Mcoln2* in CD8^+^ Teff cells. *n* = 6. **h**, Quantification of TFEB expression from western blotting in Fig. 3i and j. *n* = 3. **i**, Fluo-4 MFI of cytosolic calcium release in CD8^+^ Teff cells pretreated with Baf A1 (1 nM) for 3 hrs then treated with chloroquine (CQ, 10 μM). **j**, Lysosomal acidification of CD8^+^ Teff cells was stained with LysoSensor. *n* = 3. **k**, Fluo-4 MFI of cytosolic calcium release in CD8^+^ Teff cells treated with LA and/or LiCl. **l**, Lysosomal ^2^H concentration, determined by isotope-MS, of CD8^+^ Teff cells with or without deuterated ^2^H-acetic acid (DAC, 10 mM) or ^2^H-sulfuric acid (D_2_SO_4_, 5 mM) for 3 hrs. *n* = 3. **m**, Representation of ChIP-seq and input profiles at the *Atp6v0c* gene locus. **n**, Immunoblots of ATP6V0C in the CD8^+^ Teff cells (left). Quantification of ATP6V0C expression from western blotting (right). The control was set to 1. *n* = 3. Data are mean±s.e.m. *n* = biological replicates unless stated otherwise. *P* values were calculated using one-way ANOVA for Dunnett’s multiple comparisons test (a, b, c, e, f, g, l and n), two-way ANOVA for Tukey’s multiple comparisons test (d, h and j), ns (not significant). Source data

The above results demonstrated that LC treatment enhances lysosomal PLC/ASM activity, thus triggering PKCθ activation, MCT1 location to the IMM and the entry of LA into mitochondria. However, in mitochondria, LA had to be converted back to pyruvate for the TCA cycle. *LDHA* and *LDHB* are the two major lactate dehydrogenase genes, encoding LDHA (also known as M subunit) and LDHB (H subunit). Five isoenzymes of LDH from LDH1 to LDH5 are made by M and H subunits as 4H, 3H1M, 2H2M, 1H3M and 4M, correspondently^24^. Following LC treatment, we found that *LDHA* and *LDHB* expression was upregulated (Extended Data Fig. 5a) but LDHB was localized in the IMM of CD8^+^ T_eff_ cells (Fig. 3a and Extended Data Fig. 3b), suggesting that mitochondrial lactate dehydrogenase uses an LDH1 form to convert lactate to pyruvate. This may be in favor of the reaction, considering that LDHA has a higher affinity for pyruvate, and LDHB has a higher affinity for lactate^25^. To investigate the upregulation of LDHB expression by LC, we focused on cytosolic transcription factor EB (TFEB) because (1) TFEB is a master regulator of lysosomal function^26,27^ and (2) the above results had shown that both LA and LC exerted their effects through targeting lysosomes. We found that both TFEB expression and its nuclear localization were upregulated after LC treatment (Fig. 3b and Extended Data Fig. 5b,c), concomitant with the increased protein dephosphorylation, the active form of TFEB. By ChIP–qPCR analysis, TFEB indeed bound to the promoter of *LDHB* in LC-treated CD8^+^ T_eff_ cells (Fig. 3c). This TFEB-regulated LDHB expression was also verified by the luciferase assay (Fig. 3d). In addition, LDHB expression could not be expressed and ^13^C-labeled lactate could not be metabolized in the TCA cycles in CD8^+^ T_eff_ cells isolated from *Tfeb*^fl/fl^ *Lck*^cre^ mice (Fig. 3e,f and Extended Data Fig. 5d). Together, these results suggest that LDHB is upregulated by TFEB activation, thus cooperating with MCT1 to convert LA to pyruvate in mitochondria of LC-treated CD8^+^ T_eff_ cells.

### LC increases lysosomal pH for lysosomal Ca^2+^ release

Next, we investigated the molecular basis of TFEB activation in the LC-treated CD8^+^ T_eff_ cells. It is well known that lysosome-released Ca^2+^ acts as an important mediator to induce TFEB activation^26^. The concentration of cytosolic Ca^2+^ was elevated upon LC treatment; however, blocking mitochondrial or ER Ca^2+^ release by CGP37157 or ryanodine did not affect cytosolic Ca^2+^ in LC-treated CD8^+^ T_eff_ cells (Fig. 3g and Extended Data Fig. 5e), implying that Ca^2+^ was released from lysosomes, where Ca^2+^ can be stored or released through Ca^2+^ channels (TPC1/2 and Mcoln1/2)^26^. We found that LC treatment upregulated *Mcoln2* expression in activated CD8^+^ T_eff_ cells (Extended Data Fig. 5f). Knockdown of *Mcoln2* abrogated LC-induced Ca^2+^ release into the cytosol (Fig. 3h and Extended Data Fig. 5g), concomitant with the inactivation of TFEB and the downregulation of LDHB (Fig. 3i,j and Extended Data Fig. 5h). Then, we focused on whether LC affected lysosomal H^+^ levels, considering that lysosomal Ca^2+^ release is regulated by increased lysosomal pH^28–30^. Indeed, the increase in lysosomal pH by bafilomycin A1 and chloroquine (CQ) resulted in the release of lysosomal Ca^2+^ in LA-treated CD8^+^ T_eff_ cells (Extended Data Fig. 5i), consistent with previous reports^28,29^. In our settings, although the addition of exogenous LA, but not hydrochloric acid or sodium lactate, increased H^+^ levels in the lysosomes of CD8^+^ T_eff_ cells (Extended Data Fig. 5j), we found that LC treatment could counteract the effect of LA and increase lysosomal pH from 4.6 to 6.2 within 30 min (Fig. 3k). To determine the mechanism underlying LC-increased lysosomal pH, we added ^2^H (deuterium)-acetic acid or ^2^H-sulfuric acid to trace the proton flow into the lysosomes of CD8^+^ T_eff_ cells in the presence or absence of LC (Fig. 3l). We found that LC treatment rapidly reduced the entry of deuterium ions into the lysosomal lumen (Fig. 3m), prompting us to speculate that Li^+^ interferes with lysosomal vacuolar (V)-ATPase pumping proton. The V0 domain of V-ATPase, located in the hydrophobic lipid bilayer, is crucial for cytosolic H^+^ transport into the lysosomal lumen. Notably, Li^+^ has been reported to bind the V0 domain and induce deprotonation of the Glu139 residue in *Enterococcus hirae*^31^. In line with this, we found that Li^+^ accumulated in lysosomes after LC treatment, which, however, could be antagonized by bafilomycin A1 (Fig. 3n), which binds the c-subunit of the V0 domain. In addition, using LiCl to treat the cells, we found that Li^+^ was also concentrated in lysosomes (Fig. 3n), concomitant with increased cytosolic Ca^2+^ (Extended Data Fig. 5k). More convincingly, we demonstrated that LiCl also inhibited the pumping of deuterium ions into the lysosomes (Extended Data Fig. 5l). These results suggest that LC may rapidly interfere with proton pumping by allowing V-ATPase to translocate Li^+^ into lysosomes, thus increasing lysosomal pH. Analysis of histone lactylation by ChIP–seq revealed that the addition of exogenous LA resulted in highly enriched histone lactylation in the *Atp6v0c* gene, encoding a key subunit ATP6V0C of V-ATPase (Extended Data Fig. 4h), which, however, was erased by LC treatment (Extended Data Fig. 5m), concomitant with the downregulation of ATP6V0C expression (Extended Data Fig. 5n).

### LC treatment improves T cell-based tumor immunotherapy

**Fig. 4:**
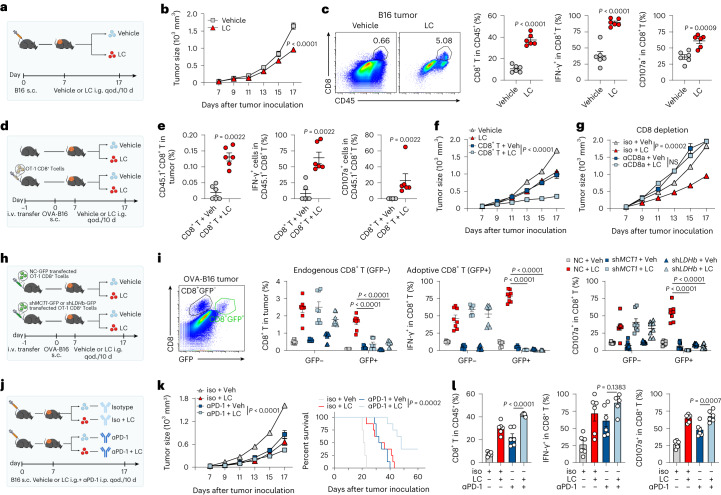
LC improves T cell-based tumor immunotherapy in mice. **a**, Schematic of experimental design for **b** and **c**. i.g., qod, intragastric administration, every other day; s.c., subcutaneous; i.v., intravenous. **b**, B16 tumor growth of C57BL/6J mice gavaged with 75 mg per kg body weight of LC once per 2 d. *n* = 7 mice. **c**, Percentage of infiltrating CD8^+^ T cells in B16 tumor, IFN-γ^+^ and CD107a^+^ cells in CD8^+^ TILs at day 18 after tumor injection in mice. *n* = 6 mice. **d**, Schematic of experimental design for **e** and **f**. **e**, Percentage of infiltrating CD45.1^+^CD8^+^ T cells in OVA-B16 tumor, IFN-γ^+^ and CD107a^+^ cells in CD45.1^+^CD8^+^ T cells at day 18 after tumor injection in mice. *n* = 6 mice. **f**, OVA-B16 tumor growth of C57BL/6J mice gavaged with LC once per 2 d. *n* = 8 mice. **g**, B16 tumor growth of C57BL/6J mice gavaged with LC once per 2 d and treated with a total of 200 μg of CD8α neutralizing antibodies once per 2 d for 10 d. *n* = 8 mice. **h**, OT-1 CD8^+^ T cells transfected with NC-GFP, sh*MCT1*-GFP or sh*LDHB-*GFP plasmids were adoptively transferred into OVA-B16 melanoma-bearing mice gavaged with LC once per 2 d. **i**, Percentage of OVA-B16-infiltrating CD8^+^GFP^+^ T cells in tumor and GFP^+^IFN-γ^+^ and GFP^+^CD107a^+^ cells in CD8^+^ TILs at day 18 after tumor injection in mice as in **h**. *n* = 8 mice. **j**, B16 tumor growth of C57BL/6J mice gavaged with LC once per 2 d and treated with a total of 10 μg of αPD-1 antibodies once per 2 d for 10 d. Created with BioRender.com. **k**, B16 tumor growth (left) and survival (right) of C57BL/6J mice. *n* = 8 mice. **l**, Percentage of infiltrating CD8^+^ T cells in B16 tumor, IFN-γ^+^ and CD107a^+^ cells in CD8^+^ TILs at day 18 after tumor injection in mice as in **j**. *n* = 6 mice. Data are presented as the mean ± s.e.m. *P* values were calculated using unpaired two-tailed Student’s *t*-test (**c**), one-way ANOVA for Dunnett’s multiple-comparisons test (**l**), two-way ANOVA for Sidak’s multiple-comparisons test (**b**), Tukey’s multiple-comparisons test (**f**, **g**, **i** and **k**, left), Mann–Whitney test (**e**) and log-rank test for survival analysis (**k** right). Source data

**Extended Data Fig. 6:**
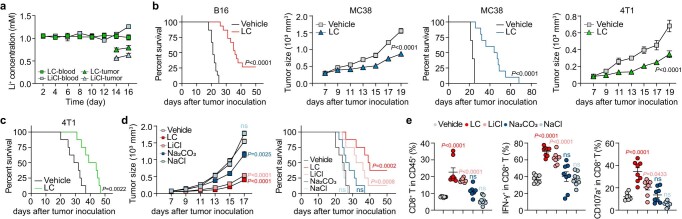
LC improves T cell-based tumor immunotherapy in mice. **a**, Li^+^ concentration in blood and tumor interstitial fluids of B16 tumor-bearing mice. *n* = 3 in LC-blood group and *n* = 4 in other groups. **b-c**, Tumor growth and survival of B16/MC38/4T1 tumor-bearing mice gavaged with or without LC once per 2 days. *n* = 15/10/8 mice in B16/MC38/4T1 model respectively. **d**, Tumor growth and survival of B16 tumor-bearing mice gavaged with LC/LiCl/Na_2_CO_3_/NaCl once per 2 days. *n* = 8 mice. **e**, Percentage of infiltrating CD8^+^ T cells in B16 tumor, IFN-γ^+^ and CD107a^+^ cells in CD8^+^ TILs at day 18 post tumor injection in mice. *n* = 8 mice. Data are presented as mean±s.e.m. *P* values were calculated using one-way ANOVA for Dunnett’s multiple comparisons test (e), two-way ANOVA for Tukey’s multiple comparisons test (d, left), Sidak’s multiple comparisons test for some tumor growth analysis (b), Log-rank test for survival analysis (b, c), and corrected by Bonferroni methods (d, right), ns (not significant). Source data

**Extended Data Fig. 7:**
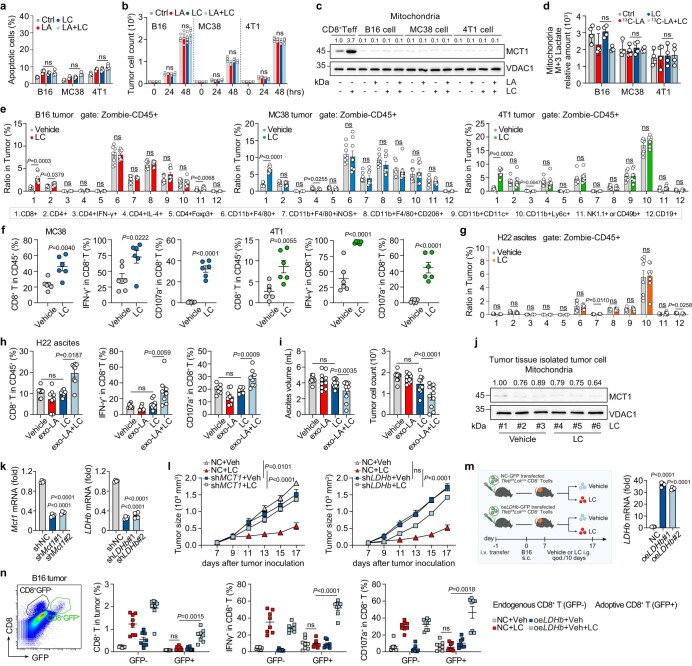
LC enhances the anti-tumor effect of CD8^+^ T cells *in vivo*. **a**, Apoptotic tumor cells treated with LA and/or LC were detected. *n* = 4. **b**, Cell count of tumor cells treated with LA and/or LC. *n* = 4. **c**, Immunoblots of MCT1 in the mitochondria of tumor cells. **d**, Relative abundance of mitochondrial M+3 lactate in tumor cells. *n* = 4. **e**, Percentage of tumor infiltrating immune subsets at day 18/20 post tumor injection in B16/MC38/4T1-bearing mice. *n* = 8 mice. **f**, Percentage of infiltrating CD8^+^ T cells in MC38/4T1 tumor, IFN-γ^+^ and CD107a^+^ cells in CD8^+^ TILs at day 20 post tumor injection in mice. *n* = 6 mice. **g**, Percentage of immune cell subsets in ascites at day 11 post tumor injection in H22 ascites mice. *n* = 8 mice. **h**, Percentage of infiltrating CD8^+^ T cells in H22 ascites, IFN-γ^+^ and CD107a^+^ cells in CD8^+^ TILs in mice gavaged with LC and intraperitoneally injected with 10 mM exogenous LA (exo-LA) once per 2 days. *n* = 8 mice. **i**, Tumor growth of H22 ascites mice gavaged with LC and intraperitoneally injected with exo-LA once per 2 days. *n* = 10 mice. **j**, Immunoblots of mitochondrial MCT1 of tumor cells from MC38 tumor-bearing mice. **k**, Silencing efficiency of sh*MCT1* and sh*LDHb* in OT-1 CD8^+^ T cells. *n* = 4. **l**, OT-1 CD8^+^ T cells transfected with NC-GFP, sh*MCT1*-GFP or sh*LDHb-*GFP plasmids were adoptively transferred into OVA-B16 bearing mice. Tumor growth was observed. *n* = 10 mice. **m**, CD8^+^ Teff cells from *Tfeb*^fl/fl^*Lck*^cre^ mice were transfected with NC-GFP or *LDHb*-overexpression plasmids (oe*LDHb*) and adoptively transferred into B16 melanoma bearing mice (left). *LDHb* expression of CD8^+^ Teff cells as in left (right). *n* = 4. **n**, Percentage of B16-infiltrating CD8^+^GFP^+^ T cells in tumor and GFP^+^IFN-γ^+^ and GFP^+^CD107a^+^ cells in CD8^+^ TILs in mice as in **m**. *n* = 8 mice. Data are mean±s.e.m. *n* = biological replicates unless stated otherwise. *P* values were calculated using unpaired two-tailed Student’s *t* test (f), one-way ANOVA for Dunnett’s multiple comparisons test (a, b, d, h, i, k, and m), two-way ANOVA for Tukey’s multiple comparisons test (l, n), Mann-Whitney test (e, g), ns (not significant). Source data

**Extended Data Fig. 8:**
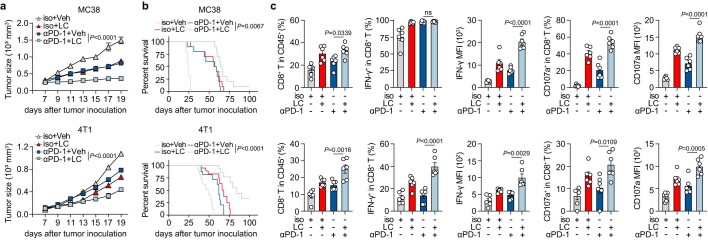
Combination of LC and PD-1 antibody results in greater anti-tumor effects. **a-b**, MC38 or 4T1 tumor growth (*n* = 9 mice in MC38 tumor, *n* = 10 mice in 4T1 mice) and survival (*n* = 10 mice) of C57BL/6J or BALB/c mice gavaged with LC once per 2 days and treated with a total of 10 μg of anti-αPD-1 antibodies once per 2 days for 10 days. **c**, Percentage of infiltrating CD8^+^ T cells in MC38 or 4T1 tumor, IFN-γ^+^ and CD107a^+^ cells in CD8^+^ TILs at day 20 post tumor injection in mice. *n* = 6 mice. Data are presented as mean±s.e.m. *P* values were calculated using one-way ANOVA for Dunnett’s multiple comparisons test (c), two-way ANOVA for Tukey’s multiple comparisons test (a), Log-rank test for survival analysis and corrected by Bonferroni methods (b), ns (not significant). Source data

Finally, we determined whether LC treatment improves T cell-based cancer immunotherapy. Based on the monitoring of lithium ions in the blood and tumor interstitial fluids (Extended Data Fig. 6a), 75 mg per kg body weight of LC (clinically equivalent dose) was selected for mouse treatment every 2 d. Using B16, MC38 and 4T1 tumor models, in which high LA levels were present (Extended Data Fig. 1a), we found that LC treatment inhibited tumor growth and prolonged mouse survival (Fig. 4a,b and Extended Data Fig. 6b,c), and LiCl had a moderate antitumor effect. However, NaCl did not generate treatment efficacy; Na_2_CO_3_ only slightly inhibited tumor growth but did not prolong mouse survival (Extended Data Fig. 6d,e), which might be attributed to the fact that CO_3_^2−^ as a moderately strong base may increase intracellular pH to facilitate glycolysis, and that LC has better pharmacokinetics than LiCl in vivo^6,32^. By conducting an in vitro culture assay, we found that LC treatment did not significantly induce B16, MC38 and 4T1 tumor cell apoptosis and proliferation (Extended Data Fig. 7a,b). Moreover, compared to CD8^+^ T_eff_ cells, tumor cells did not show MCT1 localization in mitochondria, and ^13^C-labeled lactate tracing showed negligible M + 3 lactate in the mitochondria of tumor cells (Extended Data Fig. 7c,d). Thus, LC was likely to exert its antitumor effect by targeting the antitumor immunity. By analyzing tumor-infiltrating immune cells, we found that LC treatment markedly increased the proportion of CD8^+^ T cells but did not alter the proportion of CD4^+^ regulatory T cells, CD11b^+^ myeloid cells and B cells in B16, MC38 and 4T1 tumor models (Extended Data Fig. 7e). Further analysis showed that LC treatment not only increased the proportion of tumor-infiltrating CD8^+^ T cells but also enhanced the production of interferon (IFN)-γ (effector cytokine) and CD107a (degranulation marker for perforin/granzyme B; Fig. 4c and Extended Data Fig. 7f). Then, we adoptively transferred OT-1 T cells into mice, and found that LC treatment resulted in more IFN-γ-producing OT-1 cell infiltration into OVA-B16 melanoma, concomitant with the inhibition of tumor growth (Fig. 4d–f). However, the LC-mediated antitumor effect was abrogated by CD8^+^ T cell depletion in the B16 tumor-bearing mice (Fig. 4g). In addition, using the H22 hepatocarcinoma ascites model, in which low LA levels were present, we found that LC did not promote CD8^+^ T cell activation and function in the tumor microenvironment (Extended Data Fig. 7g,h). However, injecting exogenous LA rescued the promoting effect of LC on T cells and inhibited tumor growth in the H22 ascites model (Extended Data Fig. 7h,i), suggesting that the in vivo effect of LC on CD8^+^ T cells is dependent on exogenous LA, which is consistent with the aforementioned in vitro data. To further dissect this in vivo, we isolated tumor cells from tumor tissues and found that LC treatment did not result in MCT1 localization to the mitochondria (Extended Data Fig. 7j). Transferring GFP-sh*MCT1-* or GFP-sh*LDHB-*expressing OT-1 T cells (Fig. 4h and Extended Data Fig. 7k), we found that LC-mediated tumor growth inhibition was abrogated by MCT1 or LDHB knockdown (Extended Data Fig. 7l), and LC treatment only increased the infiltration of shNC OT-1 T cells, but not sh*MCT1* or sh*LDHB* T cells, into OVA-B16 tumors and upregulated IFN-γ and CD107a expression (Fig. 4i). Consistent results were obtained from *Tfeb*^−/−^ CD8^+^ T cells, which could be augmented by *LDHB* overexpression (Extended Data Fig. 7m,n), suggesting that LC enhances CD8^+^ T cell antitumor immunity by promoting LA oxidation in vivo. Recent studies have revealed that LA is an important player in mediating resistance to PD-1 blockade^8,9,11^. Intriguingly, combination of LC and PD-1 antibody (Fig. 4j) resulted in greater tumor growth inhibition and longer survival time (Fig. 4k) and more CD8^+^ T cell infiltration and the expression of IFN-γ and CD107a than single PD-1 antibody treatment in B16 melanoma-bearing mice (Fig. 4l). Similar results were obtained from the MC38 and 4T1 tumor models (Extended Data Fig. 8a–c).

**Fig. 5:**
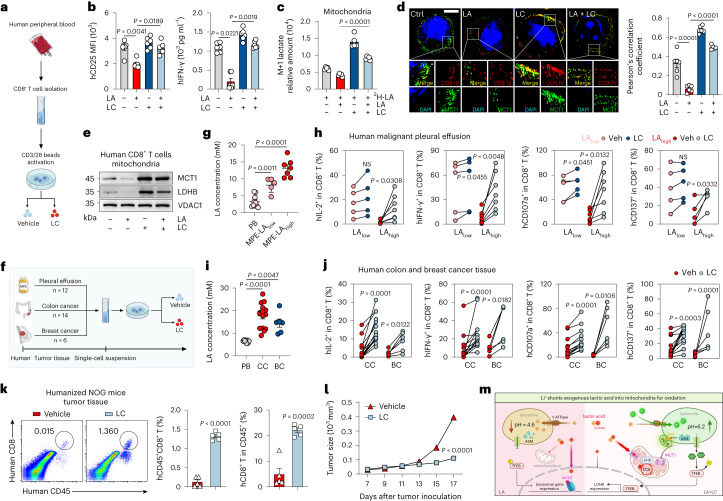
LC improves T cell function in human peripheral blood and cancer tissue. **a**, Schematic of experimental design for **b**–**e**. **b**, CD25 expression (left) and IFN-γ in supernatant (right) were detected. *n* = 6. **c**, Relative abundance of mitochondrial M + 1 lactate in human CD8^+^ T_eff_ cells treated with LA and/or LC and 2 mM ^2^H-LA. *n* = 5. **d**, Immunofluorescence staining of MCT1 and COX IV in human CD8^+^ T_eff_ cells. Scale bar, 5 μm (left). Pearson’s correlation coefficient between MCT1 and COX IV (right). *n* = 5 cells in LA + LC group and *n* = 6 cells in other groups examined over three independent experiments. **e**, Immunoblots of MCT1 and LDHB in human CD8^+^ T_eff_ cells. **f**, Schematic of experimental design for **h** and **j**. Human CD8^+^ T_eff_ cells isolated from MPE, colon cancer (CC) and breast cancer (BC) tissue were treated with LC. **g**, LA concentration in MPE was detected (LA_low _< 10 mM, *n* = 5); LA_high_ > 10 mM, *n* = 7). Peripheral blood (PB) from healthy donors (*n* = 10) was used as the control. **h**, Percentage of IL-2^+^, IFN-γ^+^, CD107a^+^and CD137^+^ cells in CD8^+^ T cells. *n* = 5 in LA_low_ and *n* = 7 i*n* LA_high_ group. **i**, LA concentration in interstitial fluid of CC (*n* = 15) and BC (*n* = 6) tissues. PB from healthy donors (*n* = 8) was used as the control. **j**, Percentage of IL-2^+^, IFN-γ^+^, CD107a^+^ and CD137^+^ cells in CD8^+^ T cells. *n* = 6 in BC group and *n* = 14 in CC group. **k**, Percentage of transferred human CD45^+^CD8^+^ T cells in A375 tumor and CD8^+^ T in CD45^+^ cells. *n* = 5 mice. **l**, A375 tumor growth of NOG mice administered with LC. *n* = 6 mice. **m**, Proposed model for LC-mediated lactate metabolism in CD8^+^ T_eff_ cells. Created with BioRender.com. Data are presented as the mean ± s.e.m. *n* = the number of patients/donors unless stated otherwise. *P* values were calculated using unpaired two-tailed Student’s *t*-test (**k**), paired two-tailed Student’s *t*-test (**h**), one-way ANOVA for Dunnett’s multiple-comparisons test (**b** right, **c**, **d**, **g** and **i**), two-way ANOVA for Sidak’s multiple-comparisons test (**l**), Kruskal–Wallis test (**b**, left) and Wilcoxon test (**j**). Source data

**Extended Data Fig. 9:**
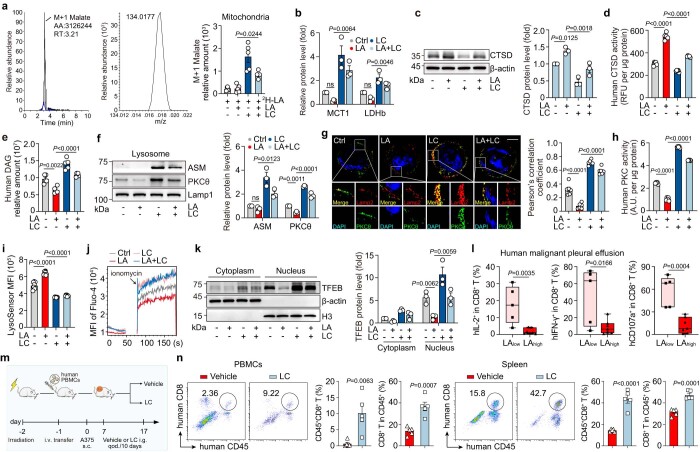
LC improves T cell function in human peripheral blood and humanized mice. **a**, Relative abundance of mitochondrial M+1 malate in human CD8^+^ Teff cells treated with LA and/or LC and 2 mM ^2^H-LA for 2 hrs. *n* = 5. **b**, Quantification of MCT1/LDHb expression from western blotting in Fig. 5e. *n* = 3. **c**, Immunoblots of CTSD in human CD8^+^ Teff cells (left). Quantification of CTSD expression from western blotting (right). The control was set to 1. *n* = 3. **d**, CTSD activity was detected in human CD8^+^ Teff cells. *n* = 5. **e**, Relative abundance of intracellular DAG in human CD8^+^ Teff cells. *n* = 5. **f**, Immunoblots of ASM and PKCθ in the lysosome of human CD8^+^ Teff cells (left). Quantification of ASM/PKCθ expression from western blotting (right). The control was set to 1. *n* = 3. **g**, Immunofluorescence staining of PKCθ and Lamp2 in human CD8^+^ Teff cells. Scale bar, 5 μm (left). Pearson’s correlation coefficient between PKCθ and Lamp2 (right). *n* = 6 cells examined over 3 independent experiments. **h**, PKC activity of human CD8^+^ Teff cells was detected. *n* = 5. **i**, Lysosomal acidification of human CD8^+^ Teff cells was stained with LysoSensor. *n* = 8. **j**, Fluo-4 MFI of cytosolic calcium release in human CD8^+^ Teff cells. **k**, Immunoblots of TFEB in human CD8^+^ Teff cells treated with LA and/or LC (left). Quantification of TFEB expression in nucleus from western blotting (right). *n* = 3. **l**, Percentage of IL-2^+^, INF-γ^+^, CD107a^+^ cells in CD8^+^ T cells derived from human malignant pleural effusion was detected by flow cytometry. The box plots show the median and first and third quartiles and the whiskers show 1.5 × interquartile range on either side. *n* = 5 in LA_low_ and *n* = 7 in LA_high_ group. **m**, Schematic of experimental design for **n**. **n**, Percentage of transferred human CD8^+^ T cells was detected in spleen and blood from humanized NOG mice treated with or without LC. *n* = 5 mice. Data are mean±s.e.m. *n* = biological replicates unless stated otherwise. *P* values were calculated using unpaired two-tailed Student’s *t* test (l, n), one-way ANOVA for Dunnett’s multiple comparisons test (a-i and k), ns (not significant). Source data

To assess LC as a potential immunotherapeutic agent applied to patients with cancer, we first determined human CD8^+^ T cells (Fig. 5a) and found that LA inhibited the activation of anti-CD3/CD28-stimulated CD8^+^ T cells derived from the peripheral blood of healthy donors; however, this could be reversed by the addition of LC (Fig. 5b). ^2^H-lactate tracing showed that LC treatment shunted lactate to the mitochondria in LA-treated human CD8^+^ T cells (Fig. 5c and Extended Data Fig. 9a). Mechanistically, LC not only facilitated human MCT1 translocation to mitochondria in a lysosomal cathepsin D-ASM-DAG-PKCθ-dependent manner (Fig. 5d,e and Extended Data Fig. 9b–h) but also upregulated human LDHB expression in a lysosomal pH-Ca^2+^-TFEB-dependent manner (Fig. 5e and Extended Data Fig. 9i–k). To translate the treatment of patients with cancer with LC, we collected malignant pleural effusion (MPE) samples from patients with lung cancer (Fig. 5f). We found that the concentration of LA was increased in the MPE (Fig. 5g) and negatively correlated with the activity of CD8^+^ T cells in the MPE (Extended Data Fig. 9l). We separated the MPEs into LA_low_ (*n* = 5) and LA_high_ (*n* = 7) groups (Fig. 5g). We found that LC treatment generated a better relief of malignant fluid-mediated suppression of CD8^+^ T cells in the LA_high_ group than in the LA_low_ group (Fig. 5h). We also collected tumor tissues from individuals with colon (*n* = 14) and breast (*n* = 6) cancer (Fig. 5f), and found that the concentration of LA in tumor tissues was increased (Fig. 5i) and LC treatment improved tumor-infiltrating CD8^+^ T cell activity (Fig. 5j). Together, these results suggest that LC exerts its antitumor effect through TIL-dependent and LA-dependent mechanisms. Moreover, using LC to treat NOG mice transplanted with human peripheral blood mononuclear cells bearing A375 human melanoma (Extended Data Fig. 9m), we found that the proportion of human CD8^+^ T cells in the tumor tissue, blood and spleen was augmented (Fig. 5k and Extended Data Fig. 9n), concomitant with the inhibition of tumor growth (Fig. 5l). Together, these results suggest that LC treatment can improve T cell-based tumor immunotherapy and has the potential to be applied to cancer patients.

## Discussion

Mammalian cells dispose of LA as a byproduct of glycolysis or as fuel in the mitochondria^15^. Because tumor cells are highly glycolytic, LA must be produced and released from tumor cells into the extracellular space despite the faster energy turnover of lactate relative to glucose^33^. However, the released LA evolutionarily becomes the accomplice for tumor cells, which effectively suppresses antitumor CD8^+^ T cell activity in the tumor microenvironment^3–5^. In this study, we elucidated the underlying molecular pathways: (1) exogenous LA induces histone lysine lactylation, upregulating the expression of lysosomal cathepsins; (2) cathepsins abrogate DAG production by targeting lysosomal ASM, thus inhibiting PKCθ activity and the subsequent phosphorylation of the MTS of MCT1; and (3) the distribution of MCT1 to the mitochondrial inner membrane is abrogated, which blocks LA oxidation in mitochondria. Based on this mechanistic illumination, we identified that LC can abrogate V-ATPase pumping protons, thus increasing lysosomal pH and rendering ASM to produce DAG for PKCθ activation and subsequent localization of MCT1 to the mitochondrial membrane. Meanwhile, increased lysosomal pH may lead to the release of stored Ca^2+^, thus activating TFEB, which transactivates LDHB to convert mitochondrial lactate into pyruvate for oxidation. Thus, LC not only triggers the escorting of MCT1 to the mitochondria but also ensures mitochondrial oxidation of the imported lactate (Fig. 5m). Although our findings support that the effect of LC in enhancing the antitumor response is mediated through lactate oxidation-dependent mechanisms, other unknown mechanisms that are independent of lactate oxidation might exist and are worthy of investigation.

Metal ions have recently been shown to play critical roles in many biological processes^34^. Transition metals, including iron, zinc, manganese, potassium, nickel, copper and cobalt, primarily participate in metabolism and redox regulation^35–38^. Metal ions are also involved in the immune system ranging from metal allergies to nutritional immunity. Magnesium can regulate CD8^+^ T cell effector functions via LFA-1 (ref. ^39^), whereas manganese can activate the cGAS–STING pathway^40^. In this study, we uncover that the lithium ion has the effect of turning waste into treasure by transforming a large amount of LA, which is a harmful waste in tumor microenvironments, into an energy source for fueling tumor-reactive CD8^+^ T cells. Our study and a new study by Jones et al. demonstrated that LA can be used to fuel CD8^+^ T cell activity^41^.

**Extended Data Fig. 10:**
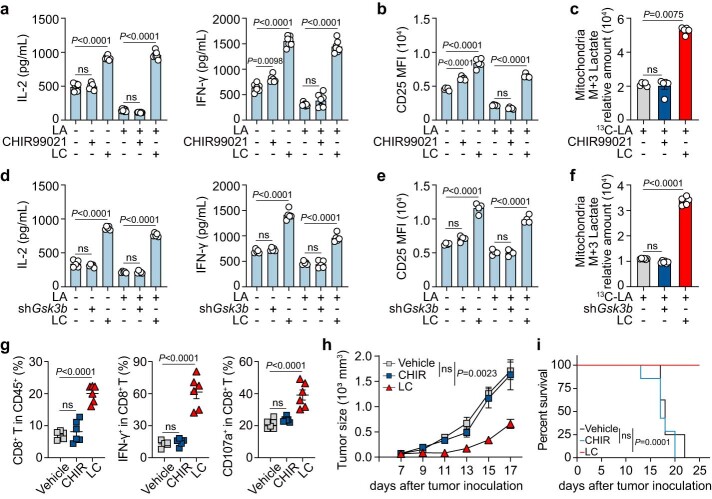
LC has GSK-independent anti-tumor function. **a**, IL-2 and IFN-γ expression, detected by ELISA, of CD8^+^ Teff cells treated with LA and LC or GSK3β inhibitor CHIR99021 (3 μM) for 24 hrs. *n* = 7. **b**, CD25 expression (MFI), analyzed by flow cytometry, of CD8^+^ Teff cells treated with LA and LC or CHIR99021. *n* = 5. **c**, Relative abundance, determined by LC-MS/MS, of mitochondrial M+3 lactate in CD8^+^ Teff cells treated with LC or CHIR99021 for 2 hrs. *n* = 5. **d**, IL-2 and IFN-γ expression, detected by ELISA, of CD8^+^ Teff cells transduced with shNC or sh*Gsk3b* and then treated with LA and/or LC. *n* = 5. **e**, CD25 expression (MFI), analyzed by flow cytometry, of CD8^+^ Teff cells transduced with shNC or sh*Gsk3b* and then treated with LA and/or LC. *n* = 4. **f**, Relative abundance, determined by LC-MS/MS, of mitochondrial M+3 lactate in CD8^+^ Teff cells transduced with shNC or sh*Gsk3b* and treated with or without LC for 24 hrs and then treated with ^13^C-lactate for 2 hrs. *n* = 6. **g-i**, C57BL/6J mice gavaged with 75 mg/kg LC or treated with CHIR99021 (16 mg/kg, *i.p*.) once per 2 days for 10 days. Percentage of infiltrating CD8^+^ T cells in B16 tumor, IFN-γ^+^ and CD107a^+^ cells in CD8^+^ TILs at day 18 post tumor injection in mice (g, *n* = 6 mice). B16 tumor growth (h, *n* = 7 mice) and survival (i, *n* = 8 mice) of C57BL/6J mice. Data are presented as mean±s.e.m. *n* = biological replicates unless stated otherwise. *P* values were calculated using one-way ANOVA for Dunnett’s multiple comparisons test (a, b, d, e, f and g), two-way ANOVA for Tukey’s multiple comparisons test (h), Kruskal-Wallis test (c), Log-rank test for survival analysis and corrected by Bonferroni methods (i), ns (not significant). Source data

LC, a first-line mood stabilizer, has been used clinically for more than 100 years, mainly to increase the inhibitory phosphorylation of glycogen synthase kinase 3β (GSK3β) on serine 9 (ref. ^42^). In addition to the inhibition of GSK3β, lithium also directly targets inositol mono-phosphatase and magnesium-dependent phosphate monoesterases and affects the intracellular homeostasis of sodium and calcium^43,44^. In this study, we found that unlike LC, the GSK3β inhibitor CHIR99021 or sh*Gsk3b* neither affected CD8^+^ T_eff_ cell function against exogenous LA nor induced the entry of lactate into mitochondria (Extended Data Fig. 10a–f). In tumor-bearing mice, GSK3β inhibition did not ameliorate tumor-infiltrating CD8^+^ T cells and antitumor treatment efficacy (Extended Data Fig. 10g–i), suggesting that LC has a GSK-independent antitumor function. Notwithstanding this, many molecules are downstream of GSK3β, including glycogen synthase, PKC, β-catenin and so on^23^. Activated GSK3β inhibits glycogenesis by phosphorylating glycogen synthase. Thus, LC likely promotes glycogenesis by inhibiting GSK3β. Our previous studies have indicated that CD8^+^ memory T cells are active in glycogenesis and glycogenolysis and the resultant glucose-6-phosphate can be shunted to the pentose phosphate pathway, thus yielding NADPH for reactive oxygen species clearance and memory longevity^45^. Based on these results, it is possible for LC to allow CD8^+^ T cells to acquire memory traits. If this is true, LC treatment will not only improve tumor-reactive CD8^+^ T cell activity but also confer cells with a high-quality antitumor response. Whether LC induces carbon flow from lactate to glycogen and confers CD8^+^ T cell memory traits is currently under investigation. In line with the findings of this study, lithium salt combination therapy has been reported to alleviate the side effects of high-lactate peritoneal fibrosis treatment and reverse the increased brain lactate during depressive episodes^16,46^, suggesting that lithium ions can regulate the catabolism of LA. However, the identification of lysosomal V-ATPase as the target of lithium ions indicates that this effect might be general in various cell types.

In summary, the data in this study clearly show that LA in tumor microenvironments, by virtue of its lactylation of histones, results in more proton pumping into the lysosomes, leading to the suppression of tumor-reactive CD8^+^ T cell activity. However, LC, by virtue of its interference with V-ATPase and blocking lysosomal acidification, results in the shunt of LA from the nucleus to mitochondria for oxidation, not only relieving the suppression but also enhancing the antitumor immunity of CD8^+^ T cells. Currently, targeting LA metabolism is emerging as a potential antitumor therapeutic option. Targeting MCT1 to cut LA uptake or LDHA to block LA generation is considered the main strategy; however, it inevitably affects the physiological functions of normal tissues. Thus, the identification of LC in this study is desirable, as it provides an alternative means of targeting LA to turn waste into a treasure for T cell-based immunotherapy.

## Methods

### Cell lines

Murine tumor cell lines B16, OVA-B16, MC38, 4T1 and H22 and human tumor cell lines A375, Hela and HEK-293T were purchased from the China Center for Type Culture Collection and cultured in RPMI 1640 or DMEM (Gibco) supplemented with 10% FBS (Gibco). All cells were cultured at 37 °C in 5% CO_2_ and routinely tested for mycoplasma.

### Mice

All animal experiments performed were approved by the Animal Care and Use Committee of Tongji Medical College. C57BL/6J, BALB/c and NOD.Cg-*Prkdc*^*scid*^*Il2rg*^*tm1Sug*^/JicCrl (NOG) mice were purchased from the Beijing Vital River Laboratory. CD45.1 C57BL/6 mice (B6.SJL-Ptprc^a^Pepc^b^/BoyJ) were obtained from Peking University Health Science Center. OT-1 TCR-transgenic mice (C57BL/6-Tg (TcraTcrb)1100Mjb/J) were donated by the laboratory of H.Z. (Sun Yat-sen University). OT-1 and CD45.1 mice were crossed to obtain OT-1 CD45.1 mice. *Tfeb*^fl/fl^ *Lck*^cre^ C57BL/6J mice were purchased from Cyagen Biosciences. All mice were kept under specific-pathogen-free conditions at the Animal Care and Use Committee of Tongji Medical College. Six- to eight-week-old female mice were used for all experiments. B16 cells (1 × 10^5^), MC38 cells (2 × 10^5^) or 4T1 cells (2 × 10^5^) were inoculated subcutaneously into C57BL/6J or BALB/c mice. For the H22 ascites model, H22 tumor cells (1 × 10^5^) were intraperitoneally injected into BALB/c mice. For the adoptive transfer model, OVA-specific OT-1 CD45.1^+^CD8^+^ T cells (1 × 10^5^) were adoptively transferred into CD45.2 recipients 24 h before OVA-B16 inoculation. For the humanized mouse tumor model, NOG mice were irradiated with 2-Gy doses of X-rays, and peripheral blood mononuclear cells (1 × 10^7^) from healthy donors were adoptively transferred into NOG mice within 24 h after irradiation. Then, A375 cells (4 × 10^5^) were inoculated subcutaneously into NOG mice. Mice were euthanized when the total tumor volume exceeded 2,000 mm^3^, as per Institutional Animal Care and Use Committee guidelines.

### Human samples

Human MPE samples from individuals with lung cancer and tumor tissue samples from individuals with breast cancer or colon cancer were obtained from the Affiliated Cancer Hospital of Zhengzhou University (Henan Cancer Hospital), the Central Hospital of Wuhan and Union Hospital, affiliated with Tongji Medical College of Huazhong University of Science and Technology. Peripheral blood was obtained from consenting healthy donors, both male and female, aged 25–30 years. All human samples used were obtained under the approval of the Ethics Committee of Huazhong University of Science and Technology (HUST-TJMU-2022 (S190)-1). The study is compliant with all relevant ethical regulations for research involving human participants.

### CD8^+^ T cell preparation

Murine CD8^+^ T cells were isolated from spleens by positive selection using naive CD8a^+^ T cell Isolation Kit (Miltenyi Biotec). Above 95% purity was confirmed by flow cytometry. Human CD8^+^ T cells were isolated from the peripheral blood of healthy donors using Human CD8^+^ T Cell Enrichment Cocktail kit (STEMCELL Technologies). Isolated CD8^+^ T cells cultured in RPMI 1640 medium containing 10% FBS, 50 μM β-mercaptoethanol (Sigma-Aldrich), 2 mM l-glutamine (Sigma-Aldrich) and 10 ng ml^−1^ IL-2 (PeproTech) were activated with mouse/human anti-CD3/CD28 Dynabeads (Thermo Fisher) for 48–72 h.

### Subcellular fractionation

Mitochondria were separated using ExKine Mitochondrion Extraction Kit (Abbkine) according to the manufacturer’s protocol. Briefly, CD8^+^ T cells (1 × 10^7^) were homogenized at 4 °C, and the homogenate was spun down at 600*g* for 10 min to collect the supernatants. The supernatants were spun down at 11,000*g* for 10 min at 4 °C to collect the mitochondria. Total mitochondrial protein contents were determined using BCA Protein Assay Kit (Thermo Fisher). Isolation of lysosomes from cells was performed using Lysosome Isolation Kit (Sigma-Aldrich) according to the manufacturer’s protocol. The nucleus was separated using Nuclei EZ Prep Kit (Sigma-Aldrich) according to the manufacturer’s protocol. The quality and purity of the isolated subcellular fraction was confirmed by immunoblot.

### LC–MS/MS analysis of metabolites

For tracing experiments, CD8^+^ T_eff_ cells were cultured with [U3]-^13^C-lactate (Sigma-Aldrich), [U6]-^13^C-glucose (Sigma-Aldrich) or ^2^D-lactate (IsoReag). To maintain the tumor extracellular pH, ^13^C-labeled lactate groups were added to an equimolar HCl titration (pH 6.6). Cells were washed with PBS and lysed in extraction solvent (80% high-performance liquid chromatography (HPLC)-grade methanol water) for 30 min at −80 °C. After centrifugation at 21,000*g* for 20 min at 4 °C, supernatant extracts were analyzed using LC–MS. The LC–MS portion of the platform was based on a HPLC (Ultimate 3000 UHPLC) system (Thermo Fisher) and a Q Exactive mass spectrometer (Thermo Fisher). Briefly, liquid chromatography was performed using an HPLC system equipped with Xbridge amide column (100 × 2.1 mm; inner diameter, 3.5 μm; Waters). The column temperature was maintained at 10 °C. Mobile phase A was 20 mM ammonium acetate and 20 mM ammonium hydroxide in water, pH 9.0, and mobile phase B was a 3:1 mixture of acetonitrile and methanol. The linear gradient was as follows: 0 min, 90% B; 1.5 min, 90% B; 5.5 min, 5% B; 8 min, 5% B; 10 min, 90% B; and 15 min, 90% B. The flow rate was 0.4 ml min^−1^. Sample volumes of 5 μl were injected for LC–MS analysis. LC–MS analysis was performed on a Q Exactive mass spectrometer equipped with a HESI probe, and the relevant parameters were: heater temperature, 120 °C; sheath gas, 30; auxiliary gas, 10; sweep gas, 3; spray voltage, 2.5 kV for the negative mode. A full scan ranging from 80 to 600 (*m/z*) was used. The resolution was set at 70,000. Data were quantified by integrating the area underneath the curve of each compound using the Xcalibur Qual browser (Thermo Fisher). Each metabolite’s accurate mass ion and subsequent isotopic ions were extracted (EIC) using a 10 ppm window. Metabolite data were analyzed by the Xcalibur software (Thermo Fisher, v.3.0).

### DAG detection

To assay the intracellular DAG, lyophilized samples were added to a 1,000 μl solution of methanol:methyltertbutylether:water (4:4:5, vol/vol), and then 20 μl of a 1:10 lipid mix lipid internal standard was added, before vortexing for 1 min. Ultrasonic crushing was performed for 32 s., followed by ultrasonic cleaning and extraction in an ice-water bath for 20 min at 4 °C and 16,000*g* centrifugation for 5 min. Next, 300 μl of supernatant was blow-dried with nitrogen, redissolved in 100 μl isopropanol:methanol (1:1, vol/vol) and vortexed for 1 min at 4 °C and 16,000*g* centrifugation for 5 min. Then, 80 μl supernatant was bottled and tested on the machine. The mobile phases in the positive and negative ion mode were an acetonitrile:water (3:2) solution containing 0.1% formic acid and 10 mm amine acetate (liquid) and isopropanol/acetonitrile (9:1) solution containing 0.1% formic acid and 10 mm amine acetate (liquid B). The flow rate was 0.3 ml min^−1^, the column temperature was 35 °C and the injection volume was 2 μl. Analysis Base File Converter software was used to convert the original data into a common format. MS-DIAL software (v.4.12) was used for preprocessing.

### Quantification of lactate

Tumor tissues were cut up with scissors, wrapped with 5-μm nylon filter paper, and stuffed (filter down) into 1.5-ml conical tubes, ensuring the tissue did not touch the bottom. Tissues were centrifuged at 1,500*g* for 2 h. The supernatants from tumor tissues, MPE and cultured cells, and cell lysates of spleens, lymph nodes and TILs were collected for the lactate concentration measurement by Lactate Assay Kit (Abcam) according to the manufacturer’s protocol.

### Measurement of intracellular acidity

Intracellular acidity was determined with pHrodo Red AM (Thermo Fisher). Cells were washed with PBS and labeled with pHrodo for 30 min. Cells were analyzed at 0–90 min after addition of 10 mM LA by flow cytometry (NovoCyte 1050 system, Agilent).

### Measurement of lysosomal pH value

LysoSensor Green DND-189 (Thermo Fisher) is typically used to qualitatively measure the pH of acidic organelles, which become more fluorescent in acidic environments and less fluorescent in alkaline environments. Cells (1 × 10^7^) were labeled with 1 μM LysoSensor in pre-warmed RPMI 1640 medium for 30 min at 37 °C and washed with PBS and immediately analyzed by flow cytometry. Quantification of lysosomal pH value was performed using ratiometric lysosomal pH dye LysoSensor Yellow/Blue DND-160 (Thermo Fisher). The pH calibration curve was generated according to the manufacturer’s protocol. Cells were labeled with 10 μM LysoSensor Yellow/Blue for 10 min at 37 °C in RPMI 1640 medium and washed with PBS. The labeled cells were treated with 10 μM valinomycin and 10 μM nigericin in 25 mM MES calibration buffer (pH 4.5–7.5) for 10 min. Quantitative comparisons were performed in a black 96-well plate, and the fluorescence was immediately measured with a microplate reader (Synergy H1, BioTek) at Ex-360/Em-460 and Ex-360/Em-528.

### Measurement of intracellular Ca^2+^

Intracellular Ca^2+^ measurement was performed as described^47^. Briefly, cells were incubated in HBSS containing 4 mM Fluo-4 AM for 1 h, washed with HBSS three times and incubated at room temperature for another 10 min. Then, 200 nM ionomycin (Biogem) was applied extracellularly and MFI of Fluo-4 was recorded within 100 s by flow cytometry (NovoCyte 1050 system, Agilent).

### Immunoblotting

Protein concentrations were measured using BCA Protein Assay Kit (Thermo Fisher). Cell lysates were prepared from T cells and separated by SDS–PAGE, and separated proteins were then transferred onto nitrocellulose membranes (Millipore). Membranes were blocked with 5% milk in TBST (TBS + 0.1% Tween-20) for 1.5 h at room temperature and incubated with primary antibodies overnight at 4 °C. Membranes were washed five times with TBST, incubated with horseradish peroxidase-conjugated secondary antibodies for 1.5 h at room temperature, washed five times with TBST again, and protein bands were visualized by ECL (Thermo Fisher). Antibody information is described in Supplementary Table 2. Quantitative analysis of protein expression was performed using ImageJ (v.1.52). The control was set to 1.

### Flow cytometric analysis

Single-cell suspensions from spleens, lymph nodes and tumor tissues were prepared. All cells were first stained with Zombie Live/Dead dye (BioLegend) at 4 °C for 30 min. To analyze surface markers, cells were stained with antibodies at 4 °C for 30 min. For intracellular cytokine staining, cells were fixed, permeabilized and labeled with intracellular cytokine antibodies. Antibody information was described in Supplementary Table 2. Data were acquired using Verse system (BD) and analyzed with FlowJo (v.10.5.3).

### Immunofluorescence microscopy

CD8^+^ T cells were fixed in 4% paraformaldehyde and adhered on glass slides using Shandon Cytospin4 Cytocentrifuge (Thermo Fisher). Cells on slides were permeabilized in 0.1% Triton X-100 and blocked with 5% BSA for 30 min at room temperature, and incubated with primary antibodies overnight at 4 °C. Cells were washed with PBS and incubated with fluorophore-conjugated secondary antibodies for 1 h at room temperature. Nuclei were stained in DAPI solution. Cells were then washed with PBS and imaged by super-resolution microscopy (DeltaVision OMX Flex, GE) or confocal microscope (SP8, Leica; LSM900, Zeiss). Pearson’s correlation coefficient was analyzed and quantified using ImageJ (v.1.52).

### PLA

Cells were fixed with 4% paraformaldehyde for 10 min, permeabilized with 0.1% Triton X-100 in PBS for 15 min and subjected to in situ PLA according to the manufacturer’s protocol (Duolink in situ Red Starter Kit Mouse/Rabbit, Sigma-Aldrich). Red fluorescent dots were analyzed and quantified using ImageJ (v.1.52).

### Gene expression analysis

Total RNA was extracted from cells with TRIzol reagent (Thermo Fisher) and reverse transcribed into cDNA by using ReverTra Ace Kit (Toyobo). cDNA was used as a template and qPCR was performed using SYBR Green (Toyobo) on a Bio-Rad CFX Connect Real-Time PCR System (v.2.0). The primer sequences were described in Supplementary Table 3.

### Viral transduction

For retroviral transduction experiments, spleen-derived CD8^+^ T cells were activated for 36 h and then transduced with concentrated retrovirus carrying pROV-U6-sh*ASM*-EF1A(S)-EGFP, pROV-U6-sh*Mcoln2*-EF1A(S)-EGFP, pLKO.1-U6-sh*Mcoln2*-Puro, pROV-U6-sh*PKCθ*-EF1A(S)-EGFP, pROV-U6-sh*MCT1*-EF1A(S)-EGFP, pROV-U6-sh*LDHB*-EF1A(S)-EGFP, pROV-U6-sh*Gsk3b*-EF1A(S)-EGFP or scramble shRNAs. In *LDHB* overexpressing settings, pROV-MSCV-IRES-EGFP plasmid was used. T cells were co-transfected with viruses at 900*g* for 2 h at 32 °C in medium containing polybrene (10 μg ml^−1^) and IL-2 (10 ng ml^−1^). EGFP or puromycin resistance was used as a marker of plasmid expression. EGFP^+^ T cells were sorted using a flow cytometer (FACS Aria II, BD) after viral transduction for 24 h. The shRNA sequences were described in Supplementary Table 4.

### Luciferase promoter activity assay

HEK-293T cells plated in 12-well plates were co-transfected with 1 μg of firefly luciferase construct containing the *LDHB* promoter and 1 μg of overexpressed *Tfeb* construct and 0.1 μg of control Renilla construct (Promega) using Lipofectamine 2000 transfection reagent (Invitrogen). After transfection, the cells were incubated for the desired time or condition, and the luciferase activity was measured using the Duo-Lite Luciferase Assay System (Vazyme) and a microplate reader (Synergy H1, BioTek). Firefly luciferase activity was normalized to Renila luciferase activity^48^.

### RNA-seq

Spleen-derived CD8^+^ T_eff_ cells were treated with 10 mM LA and/or LC for 3 h. Total RNA was extracted from cells with TRIzol according to the manufacturer’s protocol. RNA from triplicate treatment samples was purified and subjected to RNA-seq analysis using the DNBSEQ-G50 platform (BGI-Shenzhen, China). Differential gene expression analysis, KEGG analysis and heat map analysis were performed by the Dr. Tom online system (BGI-Shenzhen, China).

### ChIP–seq

DNA from chromatin immunoprecipitation was used to construct sequencing libraries following the protocol provided by the Illumina TruSeqChIP Sample Prep Set A and sequenced on an Illumina HiSeq 2000 with PE 150. Low-quality reads and adaptors were removed from the raw data using the Trimmomatic package (v.0.35), and the clean reads were mapped to the mouse genome (C57BL) using Bowtie2 (v.2.2.5) with parameters permitting <2 mismatches. Samtools (v.0.1.19) was used to remove potential PCR duplicates, and MACS software (v.1.4.2) was used to locate enriched regions to call Kla peaks by comparing reads from the IP sample with the input sample. Wig files produced by MACS software were used for data visualization with Integrative Genomics Viewer (v.2.3.88).

### ChIP–qPCR

ChIP–qPCR was performed using the ChIP Assay Kit (Active Motif) according to the manufacturer’s protocol. Briefly, CD8^+^ T_eff_ cells were fixed with 1% formaldehyde on ice to cross-link the proteins bound to the chromatin DNA. After washing, the chromatin DNA was sheared by enzymatic force to produce DNA fragments of around 200–1,000 bp. The same amounts of sheared DNA were used for immunoprecipitation using anti-TFEB or an equal amount of pre-immune IgG as control. The immunoprecipitate was then incubated with protein G magnetic beads, and the antibody-protein G magnetic beads complex was collected for subsequent reverse cross-linking. The same amount of sheared DNA without antibody precipitation was processed for reverse cross-linking and served as input control. DNA recovered from reverse cross-linking was used for qPCR. ChIP–qPCR primers for the *LDHB* promoter: primer 1 forward, 5′-CACCAGGCAGTCCTTGTAGA reverse, 5′-CATGGCATAGCTGCTCTGTG; primer 2 forward, 5′-CCAAAAATCTGTTCTCTTCTGGGT reverse, 5′-AT CACTCCAAAAGGCCTCGG.

### CTSD activity assay

CTSD activity was measured by Cathepsin D Activity Assay (Abcam) according to the manufacturer’s protocol. Briefly, CD8^+^ T_eff_ cells (1 × 10^7^) were lysed with 300 µl of chilled CD Cell Lysis Buffer and centrifuged at 21,000*g* for 5 min at 4 °C to collect the supernatant. In total, 50 µl supernatant and 52 µl reaction mix (50 µl reaction buffer and 2 µl substrate) were added to the black 96-well plate and incubated for 30 min at 37 °C. The fluorescence released upon substrate cleavage was measured using Synergy H1 microplate reader at Ex/Em = 328/460 nm. Cathepsin D activity was shown by the RFUs per microgram of protein of samples.

### PKC activity assay

PKC activity was measured by PKC Kinase Activity Assay Kit (Abcam) according to the manufacturer’s protocol. Briefly, CD8^+^ T_eff_ cells (1 × 10^6^) were lysed using native lysis buffer (Abcam) and centrifuged at 16,000*g* for 15 min at 4 °C to collect the supernatant. Diluted supernatants were pipetted to the 96-well plate pre-coated with phosphorylated substrate and the following steps were done according to the manufacturer’s protocol. The absorbance unit was recorded using a microplate reader. Protein concentrations of the samples were measured with BCA assay to normalize the PKC activity.

### Proliferation, cytokine-production and specific killing assay

For proliferation experiments, Cell Trace Violate (CTV)-labeled naive CD8^+^ T cells (2 × 10^4^) were activated with anti-CD3/CD28 beads in the presence of 10 mM LA plus LC, LiCl, Na_2_CO_3_ or NaCl for 72 h. Proliferation of responding cell populations was analyzed by CytoFLEX (Beckman Coulter). For ELISA experiments, naive CD8^+^ T cells were stimulated by anti-CD3/CD28 beads for 24 h. The same number of stimulated T cells (1 × 10^6^) per well were seeded to a 12-well plate and continually stimulated with anti-CD3/CD28 beads in the presence of different treatment. Cells were centrifuged with 400*g* for 5 min and the supernatant was detected by ELISA. For killing experiments, lymph node cells of OT-1 mice were primed by OVA-peptide (257–264) for 24 h, and then CD8^+^ T_eff_ cells were sorted and treated with 10 mM LA plus LC, LiCl, Na_2_CO_3_ or NaCl for 24 h. Then, CD8^+^ T_eff_ cells were incubated at E:T ratios (2 × 10^5^ or 4 × 10^5^ CD8^+^ T_eff_ cells to 4 × 10^4^ OVA-B6 tumor cells) for 8 h, and CD45^−^ apoptotic tumor cells were detected by flow cytometry.

### Measurement of Li^+^ concentration

Mouse blood was centrifuged at 900*g* for 15 min, and serum was collected carefully. Tumor tissues were cut up and wrapped with 5-μm nylon filter papers, and filtered down into 1.5-ml conical tubes, then centrifuged at 1,500*g* for 2 h. CD8^+^ T_eff_ cells (4 × 10^8^) were used for extraction of lysosomes by using Lysosome Isolation Kit according to the manufacturer’s protocol. Then the lysosomes were lysed with 150 μl H_2_O for Li^+^ detection. The concentration of Li^**+**^ in serum, tumor interstitial fluid and lysosomes was determined by Electrolyte Analyzer (Caretium, XI-921ET) following the manufacturer’s specifications.

### Statistical analysis

Where noted, data presented in the figures are means ± s.e.m. Multiple-group comparisons in in vivo and ex vivo assays were analyzed by Dunnett’s multiple-comparisons test (one-way ANOVA), Sidak’s multiple-comparisons test (two-way ANOVA) and Tukey’s multiple-comparisons test (two-way ANOVA). For single comparisons, an unpaired or a paired two-tailed Student’s *t*-test was used. For non-Gaussian distribution, nonparametric tests (Wilcoxon matched-pairs signed-rank test for paired single comparison, Mann–Whitney test for unpaired single comparison and Kruskal–Wallis test for unpaired multiple-group comparison) were used. All analysis was completed with GraphPad Prism software (v.8). Two-sided *P* values of less than 0.05 are considered statistically significant. In the figures, standard designations of significance are given. Specific analyses are detailed in the figure legends.

### Reporting summary

Further information on research design is available in the Nature Portfolio Reporting Summary linked to this article.

## Online content

Any methods, additional references, Nature Portfolio reporting summaries, source data, extended data, supplementary information, acknowledgements, peer review information; details of author contributions and competing interests; and statements of data and code availability are available at 10.1038/s41590-023-01738-0.

### Supplementary information

Reporting Summary

Supplementary Tables 1–4 Supplementary Table 1. MCT1 (Slc16a1) sequence; Supplementary Table 2. Regaents; Supplementary Table 3. Primer sequence; Supplementary Table 4. shRNA sequence.

### Source data

Source Data Fig. 1 Statistical source data.

Source Data Fig. 2 Statistical source data.

Source Data Fig. 2 Unprocessed fluorescence image and blots.

Source Data Fig. 3 Statistical source data.

Source Data Fig. 3 Unprocessed fluorescence image and blots.

Source Data Fig. 4 Statistical source data.

Source Data Fig. 5 Statistical source data.

Source Data Fig. 5 Unprocessed fluorescence image and blots.

Source Data Extended Data Fig./Table 1 Unprocessed blots

Source Data Extended Data Fig./Table 3 Unprocessed fluorescence image and blots.

Source Data Extended Data Fig./Table 4 Unprocessed fluorescence image and blots.

Source Data Extended Data Fig./Table 1 Statistical source data.

Source Data Extended Data Fig./Table 2 Statistical source data.

Source Data Extended Data Fig./Table 3 Statistical source data.

Source Data Extended Data Fig./Table 4 Statistical source data.

Source Data Extended Data Fig./Table 5 Statistical source data.

Source Data Extended Data Fig./Table 5 Unprocessed fluorescence image and blots.

Source Data Extended Data Fig./Table 6 Statistical source data.

Source Data Extended Data Fig./Table 7 Unprocessed blots

Source Data Extended Data Fig./Table 7 Statistical source data.

Source Data Extended Data Fig./Table 8 Statistical source data.

Source Data Extended Data Fig./Table 9 Statistical source data.

Source Data Extended Data Fig./Table 9 Unprocessed fluorescence image and blots.

Source Data Extended Data Fig./Table 10 Statistical source data.

## Data Availability

RNA-seq and ChIP–seq data that support the findings of this study (Fig. 2j,k and Extended Data Figs. 2a,b, 4h,i and 5m) have been deposited in the Science Data Bank (https://www.scidb.cn/en/s/nUzqeu/; https://www.scidb.cn/en/s/fieuyu/). Source data are provided with this paper. All other data are available in the article and Supplementary Information.
