# Supplementary material for: Lithium carbonate revitalizes tumor-reactive CD8+ T cells by shunting lactic acid into mitochondria
Source: Nat Immunol. 2024 Jan 23;25(3):552–61. doi: 10.1038/s41590-023-01738-0 (PMC10907288; doi:10.1038/s41590-023-01738-0)
Supplement: Supplementary file 1 — Reporting Summary [file 41590_2023_1738_MOESM1_ESM.pdf]

nature portfolio

Corresponding author(s): Bo Huang

Last updated by author(s): Dec 14, 2023

Reporting Summary

Nature Portfolio wishes to improve the reproducibility of the work that we publish. This form provides structure for consistency and transparency in reporting. For further information on Nature Portfolio policies, see our Editorial Policies and the Editorial Policy Checklist.

Statistics

For all statistical analyses, confirm that the following items are present in the figure legend, table legend, main text, or Methods section.

n/a	Confirmed	
☐	☒	The exact sample size (*n*) for each experimental group/condition, given as a discrete number and unit of measurement
☐	☒	A statement on whether measurements were taken from distinct samples or whether the same sample was measured repeatedly
☐	☒	The statistical test(s) used AND whether they are one- or two-sided<br>*Only common tests should be described solely by name; describe more complex techniques in the Methods section.*
☒	☐	A description of all covariates tested
☐	☒	A description of any assumptions or corrections, such as tests of normality and adjustment for multiple comparisons
☐	☒	A full description of the statistical parameters including central tendency (e.g. means) or other basic estimates (e.g. regression coefficient) AND variation (e.g. standard deviation) or associated estimates of uncertainty (e.g. confidence intervals)
☐	☒	For null hypothesis testing, the test statistic (e.g. *F*, *t*, *r*) with confidence intervals, effect sizes, degrees of freedom and *P* value noted<br>*Give P values as exact values whenever suitable.*
☒	☐	For Bayesian analysis, information on the choice of priors and Markov chain Monte Carlo settings
☒	☐	For hierarchical and complex designs, identification of the appropriate level for tests and full reporting of outcomes
☐	☒	Estimates of effect sizes (e.g. Cohen's *d*, Pearson's *r*), indicating how they were calculated

Our web collection on statistics for biologists contains articles on many of the points above.

Software and code

Policy information about availability of computer code

Data collection	-qPCR was performed on a Bio-Rad CFX Connect Real-Time PCR System (v.2.0).<br>-Immunofluorescence data were captured by super-resolution microscopy (DeltaVision OMX Flex, GE) and confocal microscope (SP8, Leica; LSM900, Zeiss). Pearson's correlation coefficient was analyzed and quantified using Image J (v.1.52).<br>-Flow cytometric data were captured using BD Verse (BD Biosciences), FACS Aria II (BD Biosciences), NovoCyte 1050 system (Agilent) and CytoFLEX (Beckman Coulter).<br>-Metabolism analysis data were captured using the Xcalibur™ software (Thermo Fisher, v.3.0).<br>-Immunoelectron microscopy data were viewed on a JEOL 1200EX transmission electron microscope (JEOL USA, Inc.).
Data analysis	GraphPad Prism software (v.8.0) was used for analysis of in vivo and in vitro phenotype assays and for graph production. FlowJo (v.10.5.3) was used for analysis of flow cytometry data. Image J (v.1.52) was used for visualization and presentation of fluorescence imaging. IGV_2.3.88 was used for histone lactylation analysis. For further details on these Methods and specific references please see Methods section.

For manuscripts utilizing custom algorithms or software that are central to the research but not yet described in published literature, software must be made available to editors and reviewers. We strongly encourage code deposition in a community repository (e.g. GitHub). See the Nature Portfolio guidelines for submitting code & software for further information.

Data

Policy information about availability of data

All manuscripts must include a data availability statement. This statement should provide the following information, where applicable:

- Accession codes, unique identifiers, or web links for publicly available datasets
- A description of any restrictions on data availability
- For clinical datasets or third party data, please ensure that the statement adheres to our policy

RNA-seq and ChIP-seq data that support the findings of this study (Extended Data Fig. 2a, 2b and Fig. 2j, 2k and Extended Data Fig. 4h, 4i, 5m) have been deposited in the science data bank (https://www.scidb.cn/en/s/nUzqeu; https://www.scidb.cn/en/s/fieuyu). The data supporting the findings of this study are available within the paper and the Extended Data figures. Source data are provided with this paper.

Human research participants

Policy information about studies involving human research participants and Sex and Gender in Research.

Reporting on sex and gender	The healthy volunteers included men and women, aged 25-30 years, and patients with malignant pleural effusion, breast cancer, colon cancer included men and women, aged 46-72 years.
Population characteristics	The study population was comprised peripheral blood, tumor tissue of breast cancer, colon cancer and malignant pleural effusion. Peripheral blood was obtained from consenting healthy donors (aged 25-30). Tumor tissue was acquired after surgery and malignant pleural effusion was acquired by drainage (aged 46-72 years).
Recruitment	Human malignant pleural effusion from lung cancer patients and tumor tissue from breast cancer and colon cancer patients were obtained from the Affiliated Cancer Hospital of Zhengzhou University (Henan Cancer Hospital), the Central Hospital of Wuhan and Union Hospital, affiliated with Tongji medical college of Huazhong University of Science and Technology. Peripheral blood was obtained from consenting healthy donors.
Ethics oversight	All of the human samples used in this study were obtained under the approval of the Ethics Committee of the Huazhong University of Science and Technology, and informed consent was obtained from all of the participants. The study complies with all relevant ethical regulations regarding research involving human participants.

Note that full information on the approval of the study protocol must also be provided in the manuscript.

Field-specific reporting

Please select the one below that is the best fit for your research. If you are not sure, read the appropriate sections before making your selection.

☒ Life sciences ☐ Behavioural & social sciences ☐ Ecological, evolutionary & environmental sciences

For a reference copy of the document with all sections, see nature.com/documents/nr-reporting-summary-flat.pdf

Life sciences study design

All studies must disclose on these points even when the disclosure is negative.

Sample size	Group sizes for experiments were selected based on previously published studies (Nat Immunol. 2023 Dec;24(12):2042-2052; Nature cell biology, 2020 ;22(1):18-25) and further enlarged upon the request of the reviewer in the revision. These sample sizes are sufficient to allow for the determination of statistical significance between groups and minimized the number of animals or replicates needed for each experiment. The group size for each individual experiment is mentioned in the figure legend.
Data exclusions	No data were excluded from analysis.
Replication	Replicates were used in all experiments as noted in text, figure legends and methods. All experiments presented for which replication was attempted were successfully replicated.
Randomization	Mice were placed into experimental group by nature of their genotype and/or if receiving treatment were randomized within a genotype. For experiments not involving mice, cells were randomized into experimental groups.
Blinding	The tumor measurement was performed with cage labels blinded for treatments. The treatments were performed after the tumor measurement. For in vitro experiments, experiments execution and analysis was performed by different people in the lab.

Reporting for specific materials, systems and methods

We require information from authors about some types of materials, experimental systems and methods used in many studies. Here, indicate whether each material, system or method listed is relevant to your study. If you are not sure if a list item applies to your research, read the appropriate section before selecting a response.

Materials & experimental systems

n/a	Involved in the study
☐	☒ Antibodies
☐	☒ Eukaryotic cell lines
☒	☐ Palaeontology and archaeology
☐	☒ Animals and other organisms
☒	☐ Clinical data
☒	☐ Dual use research of concern

Methods

n/a	Involved in the study
☐	☒ ChIP-seq
☐	☒ Flow cytometry
☒	☐ MRI-based neuroimaging

Antibodies

Antibodies used

For flow cytometry analysis: Rat anti-mouse CD8a, Brilliant Violet 421 (53-6.7, Cat# 100738, Lot# B358296, Biolegend, 1:100), Rat anti-mouse CD107a, APC (1D4B, Cat# 121614, Lot# B338818, Biolegend, 1:100), Rat anti-mouse CD45, FITC (30-F11, Cat# 103108, Lot# B363912, Biolegend, 1:100), Rat anti-mouse IFN-γ, APC (XMG1.2, Cat# 505810, Lot# B354911, Biolegend, 1:100), Mouse anti-mouse CD45.1, FITC (clone A20, Cat# 110706, B334476, Biolegend, 1:100), Rat anti-mouse F4/80, APC (BM8, Cat# 123116, Lot# B379316, Biolegend, 1:100), Armenian Hamster anti-mouse CD11c, Brilliant Violet 421 (B418, Cat# 117330, Lot# B366519, Biolegend, 1:100), Rat anti-mouse/human CD11b, PerCP/Cyanine5.5 (M1/70, Cat# 101228, Lot# B375904, Biolegend, 1:100), Mouse anti-mouse NK-1.1, PE (PK136, Cat# 108707, Lot# B343834, Biolegend, 1:100), Rat anti-mouse CD49b (pan-NK cells),PE (DX5, Cat#108907, Biolegend, 1:100), Rat anti-Nos2 (iNOS), PE (W16030c, Cat# 696806, Lot# B356090, Biolegend, 1:100), Rat anti-mouse CD19, APC (6D5, Cat# 115511, Lot# B372547, Biolegend, 1:100), Rat anti-mouse Ly-6C, APC (HK1.4, Cat# 128016, Lot# B373764, Biolegend, 1:100), Rat anti-mouse CD4, Brilliant Violet 421 (RM4.5, Cat# 100544, Lot# B375516, Biolegend, 1:100), Rat anti-mouse CD206 (MMR), PE (C068C2, Cat# 141706, Lot# B369244, Biolegend, 1:100), Mouse anti-mouse/rat/human FOXP3, Alexa Fluor 647 (150D, Cat# 320014, Lot# B370699, Biolegend, 1:100), Mouse anti-human CD45, FITC (HI30, Cat# 304038, Lot# b356107, Biolegend, 1:100), Mouse anti-human IFN-γ, APC (4S.B3, Cat# 502512, Lot# B351305, Biolegend, 1:100), Mouse anti-human CD8a, Brilliant Violet 421 (RPA-T8, Cat# 301036, Lot# B358095, Biolegend, 1:100), Mouse anti-human CD25, APC (BC96, Cat# 302610, Biolegend, 1:100), Mouse anti-human CD107a, APC (H4A3, Cat# 328620, Lot# B355169, Biolegend, 1:100), Mouse anti-human CD137, PE (4B4-1, Cat# 309804,Lot# B343311, Biolegend, 1:100), Rat anti-human IL-2, APC (MQ1-17H12, Cat# 500310, Lot# B335968, Biolegend, 1:100),Rat anti-mouse IL-4, PE/Dazzle™ 594 (11B11, Cat# 504131, BioLegend, 1:100). For western blot/ Immunofluorescence analysis: Rabbit anti-human MCT1 mAb (E7F6Y, Cat# 36768, Cell Signaling Technology, 1:1000 for WB, 1:50 for IF), Rabbit anti-human/mouse MCT1 pAb (Polyclonal, Cat# 20139-1-AP, Proteintech, 1:1000 for WB, 1:50 for IF), Mouse anti-human MCT1 mAb (P14612, Cat# MA5-18288, Invitrogen, , 1:1000 for WB, 1:50 for IF), Mouse anti-human/mouse LAMP2 mAb (2D3B9, Cat# 66301-1-Ig, Proteintech, , 1:1000 for WB, 1:50 for IF), Rabbit anti-human/mouse PKCθ mAb (E1I7Y, Cat# 13643, Cell Signaling Technology, 1:1000 for WB, 1:50 for IF), Mouse anti-human/mouse LDHB mAb (5E9G1, Cat# 66425-1-Ig, Proteintech, 1:1000 for WB, 1:50 for IF), Rabbit anti-human/mouse LDHB-Specific pAb(Polyclonal, Cat# 19988-1-AP, Proteintech, 1:1000 for WB, 1:50 for IF), Rabbit anti-human TFEB mAb (D2O7D, Cat# 37785, Cell Signaling Technology, 1:1000 for WB, 1:50 for IF), Rabbit anti-mouse TFEB pAb (Polyclonal, Cat# 13372-1-AP, Proteintech, 1:1000 for WB, 1:50 for IF), Mouse anti-human/mouse COX IV mAb(2A7B2, Cat# 66110-1-Ig, Proteintech, 1:1000 for WB, 1:50 for IF), Donkey anti-rabbit IgG H&L, Alexa Fluor 488 (Cat# A-21206, Invitrogen, 1:200), Donkey anti-mouse IgG H&L, Alexa Fluor 594 (Cat# A-21203, Invitrogen, 1:200), Rabbit anti-human/mouse VDAC1 mAb (D73D12, Cat# 4661, Cell Signaling Technology, 1:1000), Rabbit anti-human/mouse Histone H3 mAb (D1H2, Cat# 4499, Cell Signaling Technology, 1:1000), Mouse anti-human/mouse β-Actin mAb (8H10D10, Cat# 3700, Cell Signaling Technology, 1:10000), Rabbit anti-human/mouse L-Lactyl Lysine mAb (9H1L6, Cat# PTM-1401RM, PTMBIO, 1:1000), Rabbit anti-human/mouse LAMP1 pAb (Polyclonal, Cat# ab24170, Abcam, 1:1000), Rabbit anti-human/mouse Cathepsin D pAb (Polyclonal, Cat# 21327-1-AP, Proteintech, 1:1000), Rabbit anti-human/mouse Cathepsin G mAb (ARC54792, Cat# A22048, ABclonal, 1:1000), Rabbit anti-human/mouse ASM pAb (Polyclonal, Cat# 14609-1-AP, Proteintech, 1:1000), Rabbit anti-human/mouse Cathepsin E pAb (Polyclonal, Cat# A2678, ABclonal, 1:1000), Rabbit anti-human/mouse ATP6V0C pAb (Polyclonal, Cat# A16350, ABclonal, 1:1000), Rabbit anti-human Na+/K+-ATPase α1 mAb (D4Y7E, Cat# 23565, Cell Signaling Technology, 1:1000).
For in vivo: Ultra-LEAF™ Purified anti-mouse PD-1 Antibody (29F.1A12, Cat# 135250, Biolegend), Ultra-LEAF™ Purified anti-mouse CD8a Recombinant Antibody (QA170A07, Cat# 155011, Biolegend).

Validation

All antibodies are commercial available and were validated on the manufacturers website. For flow cytometry, antibodies were validated as noted on manufacturer's website, and most of antibodies specificity was confirmed in the literature. Rat anti-mouse CD8a, Brilliant Violet 421; Rat anti-mouse CD107a, APC; Rat anti-mouse CD45, FITC ; Rat anti-mouse IFN-γ, APC; Mouse anti-mouse CD45.1, FITC; Rat anti-mouse F4/80, APC; Armenian Hamster anti-mouse CD11c, Brilliant Violet 421; Rat anti-mouse/human CD11b, PerCP/Cyanine5.5; Rat anti-Nos2 (iNOS), PE; Rat anti-mouse CD19, APC; Rat anti-mouse Ly-6C, APC; Rat anti-mouse CD4, Brilliant Violet 421; Rat anti-mouse CD206 (MMR), PE; Mouse anti-mouse/rat/human FOXP3, Alexa Fluor 647; Mouse anti-human CD45, FITC; Mouse anti-human IFN-γ, APC; Mouse anti-human CD8a, Brilliant Violet 421; Mouse anti-human CD25, APC; Mouse anti-human CD107a, APC; Mouse anti-human CD137, PE were demonstrated to work for flow cytometry by previous publications of others and our own groups in the species tested. For western blot and immunofluorescence, all antibodies were used as validated by the manufacturer for their specific assay according to their datasheet. In addition, the stainings were consistent with the predicted cellular localization of the protein.

Eukaryotic cell lines

Policy information about cell lines and Sex and Gender in Research

Cell line source(s)

Murine tumor cell lines B16, OVA-B16 (melanoma), 4T1 (breast cancer), MC38 (colon cancer) and H22 (hepatocarcinoma);

Cell line source(s)	human tumor cell line A375 (melanoma); Hela (cervical carcinoma cell line) and human embryonic kidney cell line HEK-293T were purchased from the China Center for Type Culture Collection
Authentication	None of the cell lines were independently authenticated.
Mycoplasma contamination	Our cell lines are routinely tested for mycoplasma. None of the cell lines used in this study have tested positive for mycoplasma.
Commonly misidentified lines (See ICLAC register)	None.

Animals and other research organisms

Policy information about studies involving animals; ARRIVE guidelines recommended for reporting animal research, and Sex and Gender in Research

Laboratory animals	All mice used in animal experiments bred in specific pathogen-free environment, maintained at room temperature 20-26°C with relative humidity 30-70. Mice were maintained on a 12-h light/12-h dark cycle. All experimental animals were fed with transgenic lab mice diet (Xie tong sheng wu, SAS9126).All animal experiments performed were approved by the Animal Care and Use Committee of Tongji Medical College. C57BL/6J, BALB/c and NOD.Cg-PrkdcscidIl2rgtm1Sug/JicCrl (NOG) mice were purchased from the Beijing Vital River Laboratory. CD45.1 C57BL/6 mice (B6.SJL-PtprcaPepcb/BoyJ) were obtained from Peking University Health Science Center. OT-1 TCR-transgenic mice (C57BL/6-Tg (TcraTcrb)1100Mjb/J) were donated by H. Zhang's laboratory (Sun Yat-sen University). OT-1 and CD45.1mice were crossed to obtain OT-1 CD45.1 mice. Tfebfl/fl Lckcre C57BL/6J mice were purchased from Cyagen Biosciences Inc.All experiments were performed on 6-8-week-old female animals.
Wild animals	No wild animals were used for this study.
Reporting on sex	All experimental animals were female mice.
Field-collected samples	No field samples were collected for this study.
Ethics oversight	All animal experiments were conducted in accordance with a protocol approved by the Animal Care and Use Committee Tongji medical College.

Note that full information on the approval of the study protocol must also be provided in the manuscript.

ChIP-seq

Data deposition

☒ Confirm that both raw and final processed data have been deposited in a public database such as GEO.

☒ Confirm that you have deposited or provided access to graph files (e.g. BED files) for the called peaks.

Data access links *May remain private before publication.*	https://www.scidb.cn/en/s/fieuyu
Files in database submission	.bed and .fastq files are provided.
Genome browser session (e.g. UCSC)	No longer applicable.

Methodology

Replicates	There are no biological replicates.
Sequencing depth	All ChIP-seq datasets were sequenced with 2*150 bp paired-end reads. The total number of reads for each experiments is 70M.
Antibodies	ChIP-seq was performed using an anti-human/mouse L-Lactyl Lysine mAb (9H1L6, Cat# PTM-1401RM, PTMBIO).
Peak calling parameters	Enriched peaks were discovered using MACS2 with a FDR cutoff of 0.01.
Data quality	ChIP-seq data were assessed by FastQC and the ends of sequencing reads were trimmed. Data were aligned by Bowtie2 to the mouse genome. UCSC genes from the iGenome mouse mm10 assembly were used for gene annotation. MACS2 was used to identify peaks of enrichment with a FDR cutoff of 0.01.
Software	Fastqc version 0.11.8; trimmomatic version 0.39; Alignment: Bowtie2 version 2.3.5; Peak calling: macs2 version 2.1.2; Annotation of peaks: homer version 4.9.1

Flow Cytometry

Plots

Confirm that:

☒ The axis labels state the marker and fluorochrome used (e.g. CD4-FITC).

☒ The axis scales are clearly visible. Include numbers along axes only for bottom left plot of group (a 'group' is an analysis of identical markers).

☒ All plots are contour plots with outliers or pseudocolor plots.

☒ A numerical value for number of cells or percentage (with statistics) is provided.

Methodology

Sample preparation	Preparation of single cell suspension from spleen and lymph nodes: spleen or lymph nodes were harvested into a tissue culture dish with 5 mL PBS containing 0.5% FBS, then single cell suspensions were obtained by press with the plunger of a 5-mL syringe. Pass cell suspension through the cell strainer to eliminate clumps and debris, then centrifuge at 500 g for 5 minutes at 4°C to harvest the cell pellet, and repeat the wash step one time. Finally, re-suspend the cell pellet in PBS to the final concentration of 1 x 10^7 cells/mL and used for cell staining. Tumors from various treatment groups of mice were digested with collagenase and hyaluronidase for 1hr at 37°C,and homogenized with semi-frosted slides. After lysis of RBC, Cells are activated by cell activation cocktail for 4 hrs. and then filtered to obtain a single cell suspension for staining. For patients with malignant pleural effusion, 400 g centrifugation was performed for 10 minutes and then staining was performed to detect the phenotype after 48 hours-treatment.
Instrument	BD FACSVerse Flow cytometer
Software	FlowJo software
Cell population abundance	The single cell suspension from spleen were transduced with concentrated retrovirus carrying pROV-U6-shASM-EF1A(S)-EGFP, pROV-U6-shMcoln2-EF1A(S)-EGFP, pLKO.1-U6-shMcoln2-Puro, pROV-U6-shPKCθ-EF1A(S)-EGFP, pROV-U6-shMCT1-EF1A(S)-EGFP, pROV-U6-shLDHb-EF1A(S)-EGFP, pROV-U6-shGsk3b-EF1A(S)-EGFP or scramble shRNAs. In LDHb overexpressing settings, pROV-MSCV-IRES-EGFP plasmid was used. EGFP+ cells were sorted in BD FACS Aria II with purity of priority.
Gating strategy	For all experiments, debris was first excluded by a morphology gate based on FSC-A and SSC-A. Then, non-singlets were eliminated from analysis by a single cell gate based on FSC-H and FSC-A. GFP+,FITC+, PE+, BV421+, V500+ or APC+ cells were gated and isotype control antibodies were used after appropriate compensation using single-stained compensation controls.

☒ Tick this box to confirm that a figure exemplifying the gating strategy is provided in the Supplementary Information.
