# Supplementary figures and images for: Lithium carbonate revitalizes tumor-reactive CD8+ T cells by shunting lactic acid into mitochondria
Source: Nat Immunol. 2024 Jan 23;25(3):552–61. doi: 10.1038/s41590-023-01738-0 (PMC10907288; doi:10.1038/s41590-023-01738-0)

**Figure 2a**

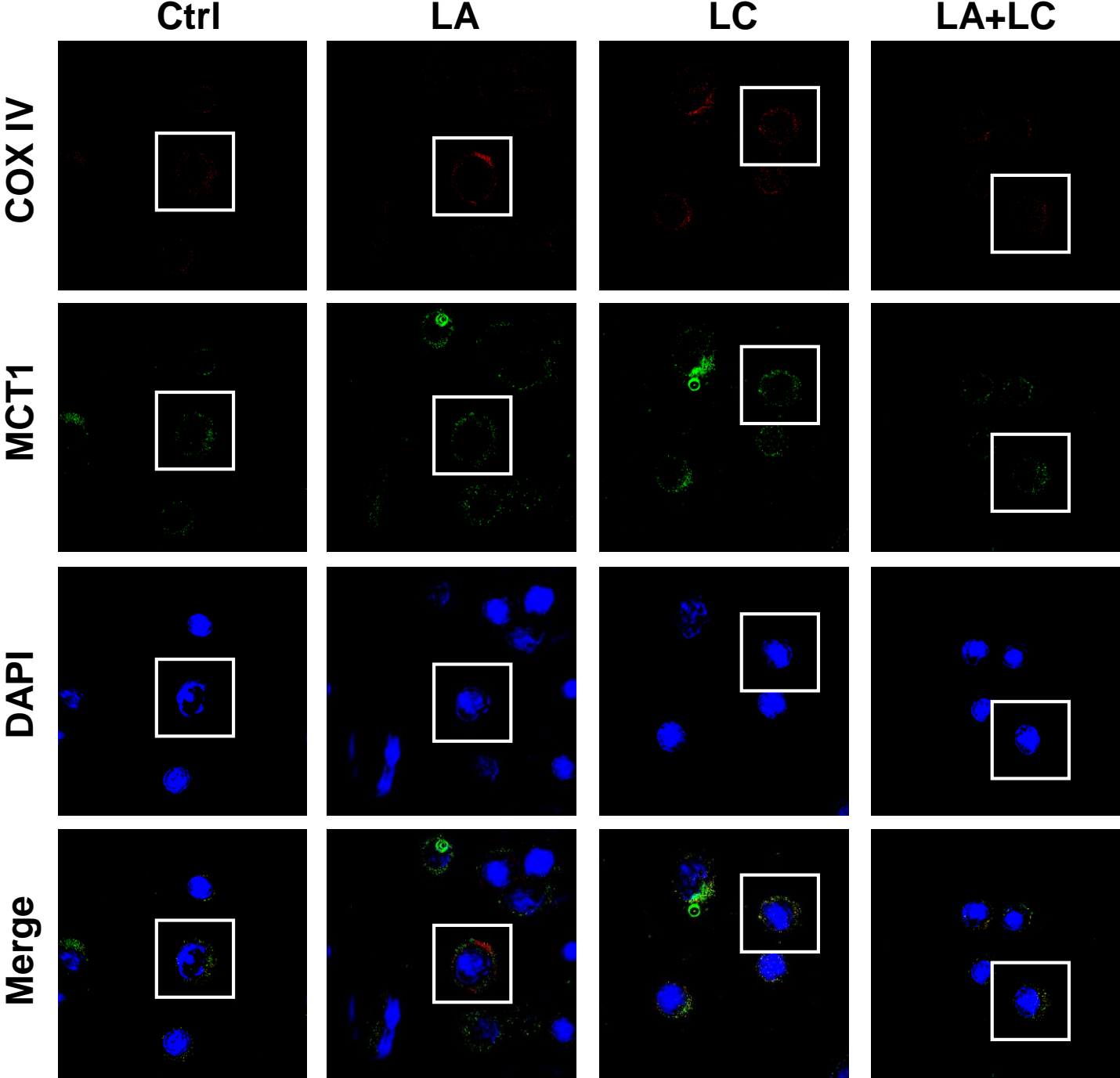

**Figure 2b**

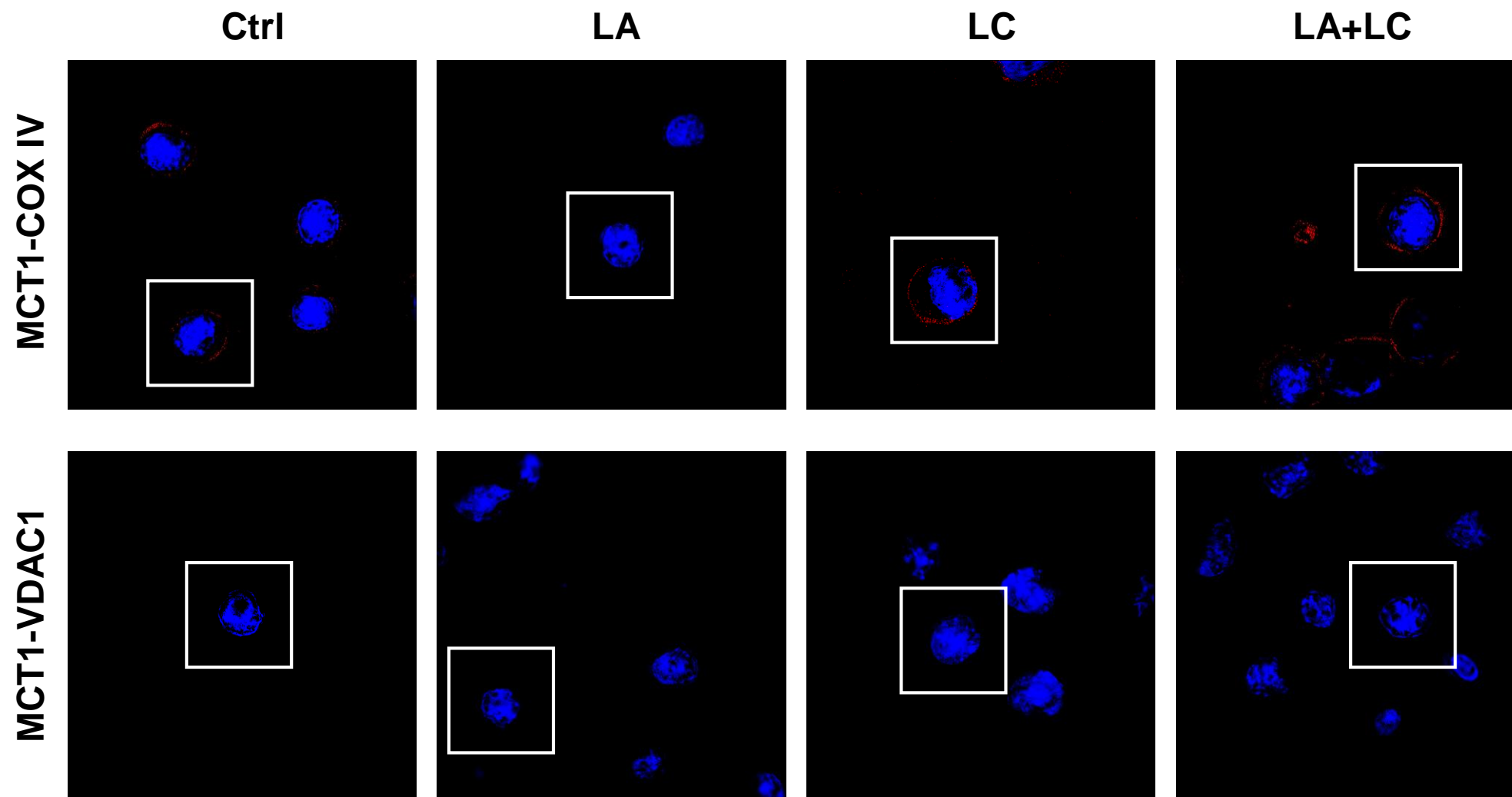

Figure 2g

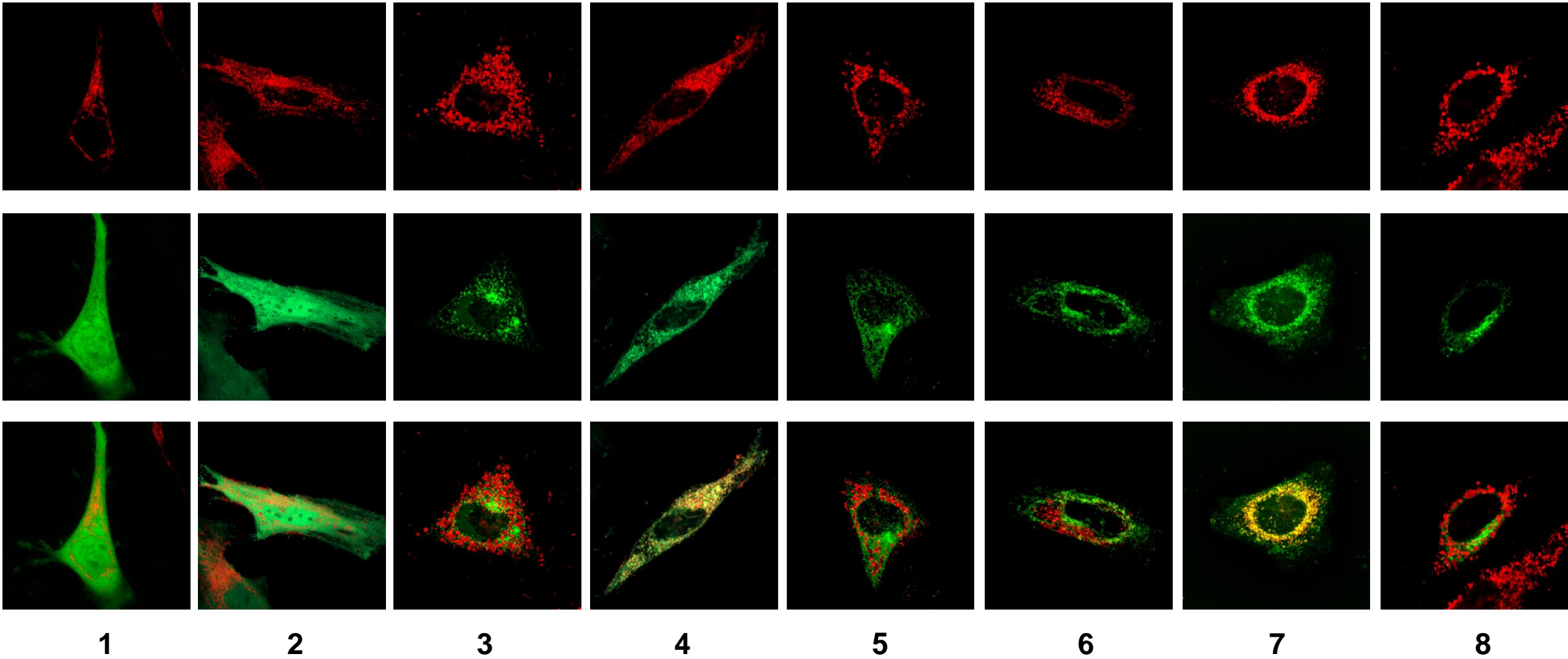

[illegible]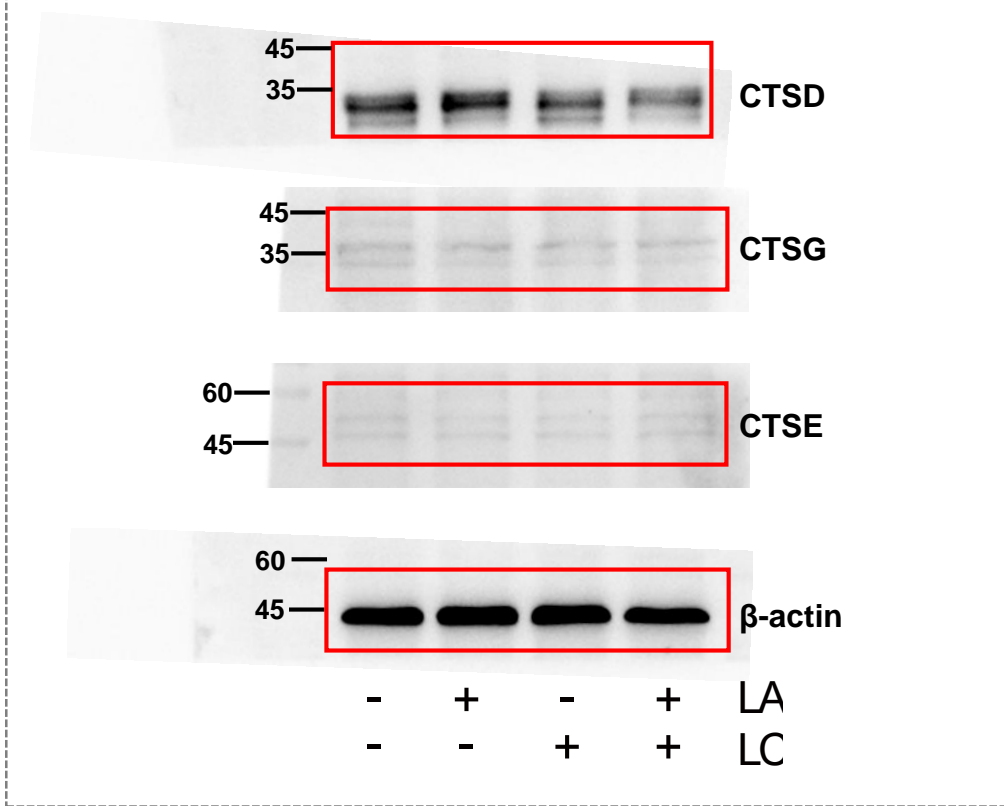

Supplement: Supplementary file 5 — Unprocessed fluorescence image and blots. [file 41590_2023_1738_MOESM5_ESM.pdf]

Figure 3a

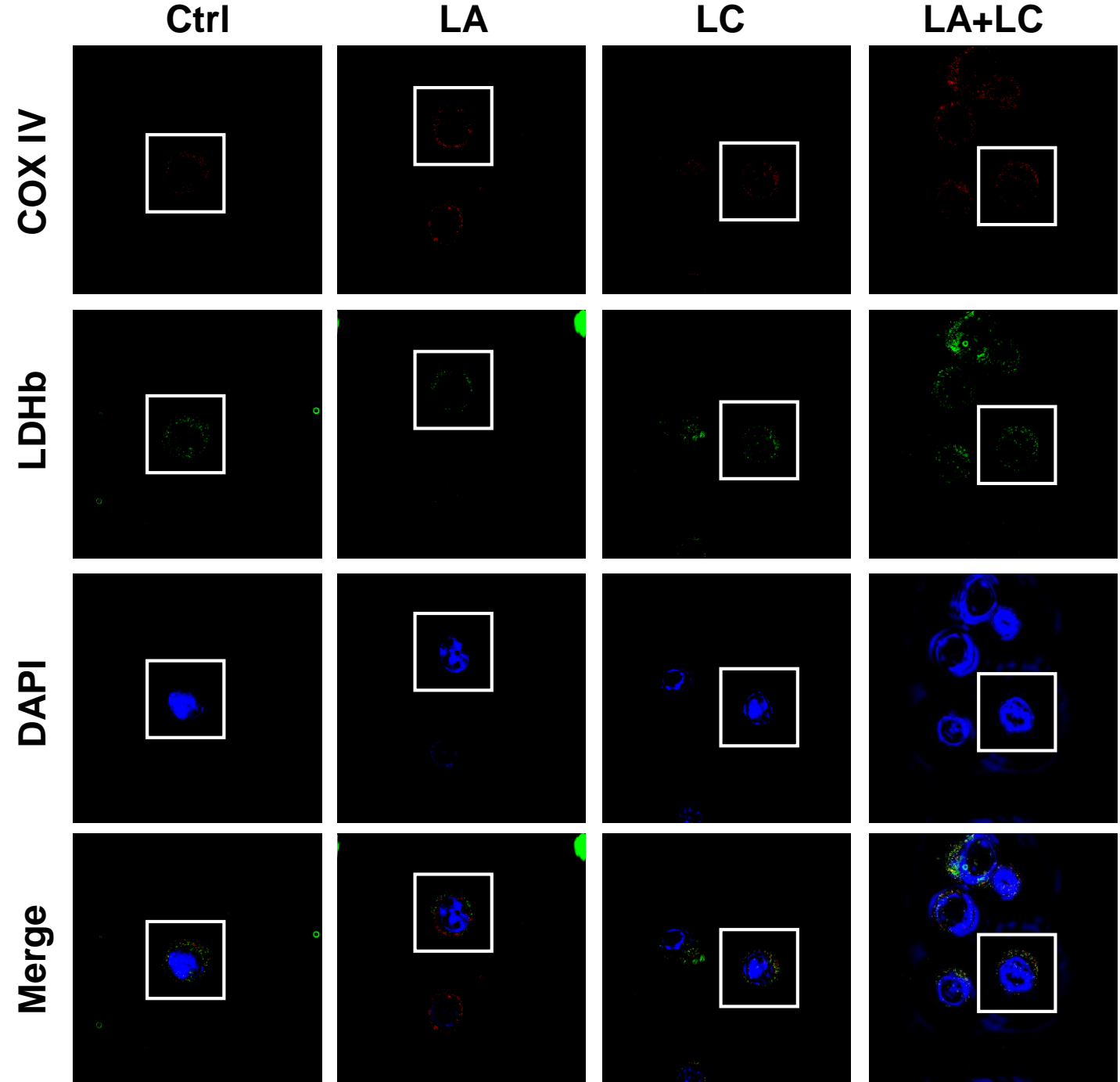

**Figure 3b**

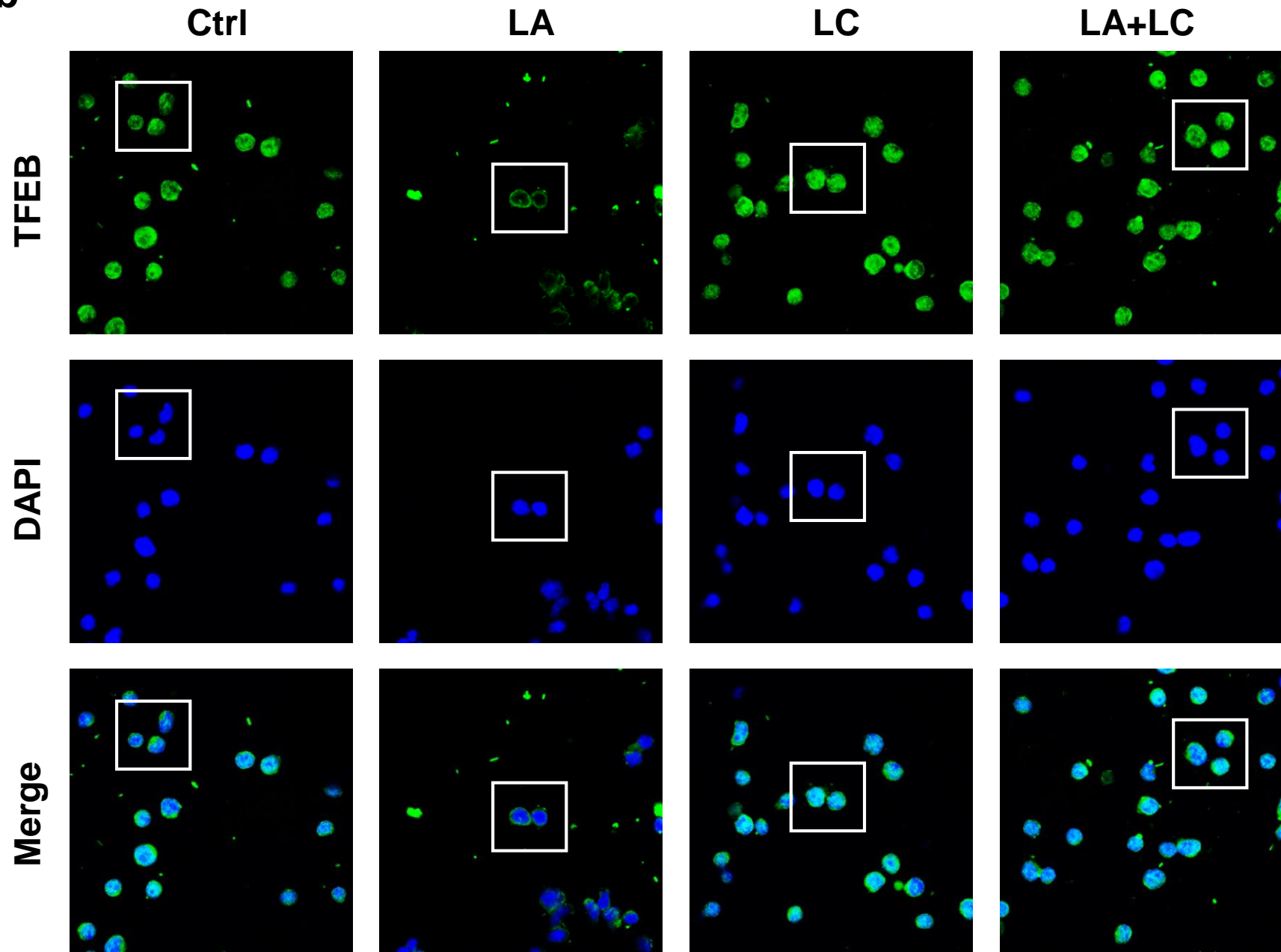

**Figure 3e**

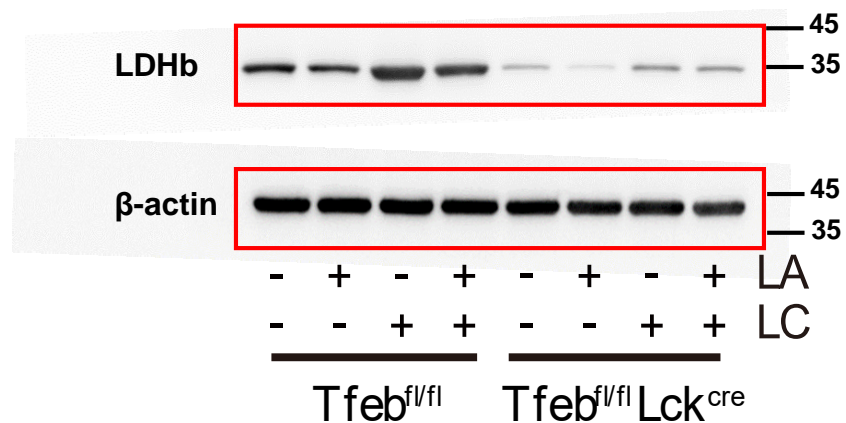

**Figure 3i**

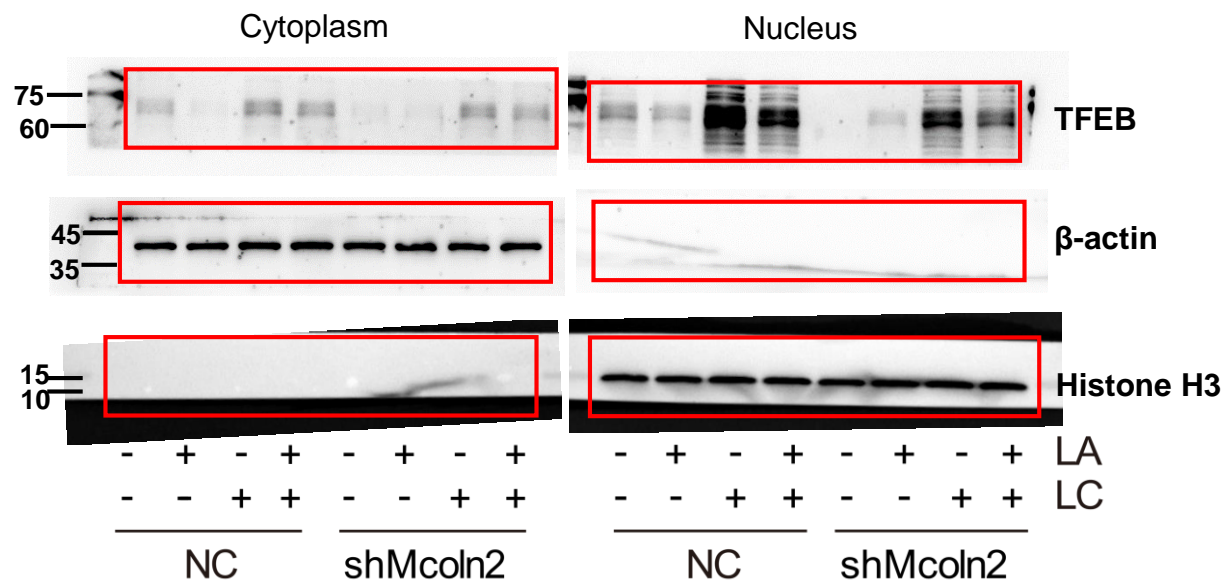

**Figure 3j**

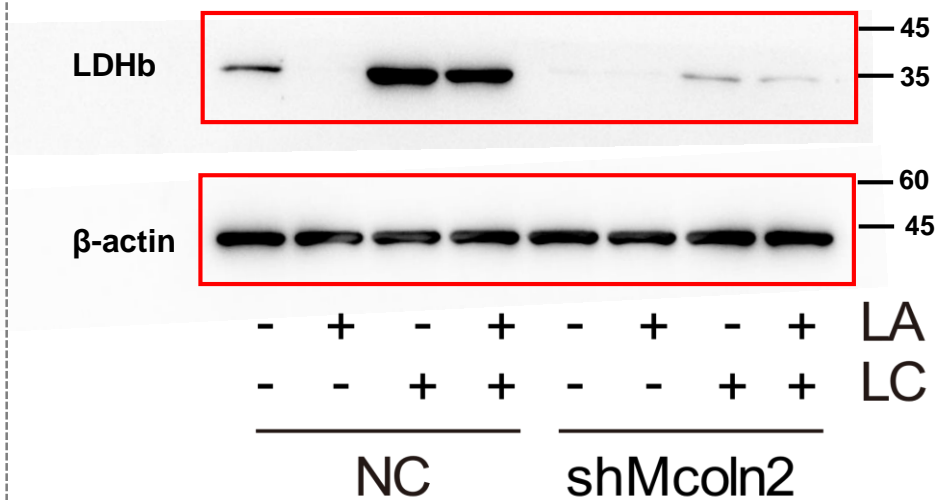

Supplement: Supplementary file 7 — Unprocessed fluorescence image and blots. [file 41590_2023_1738_MOESM7_ESM.pdf]

**Figure 5d**

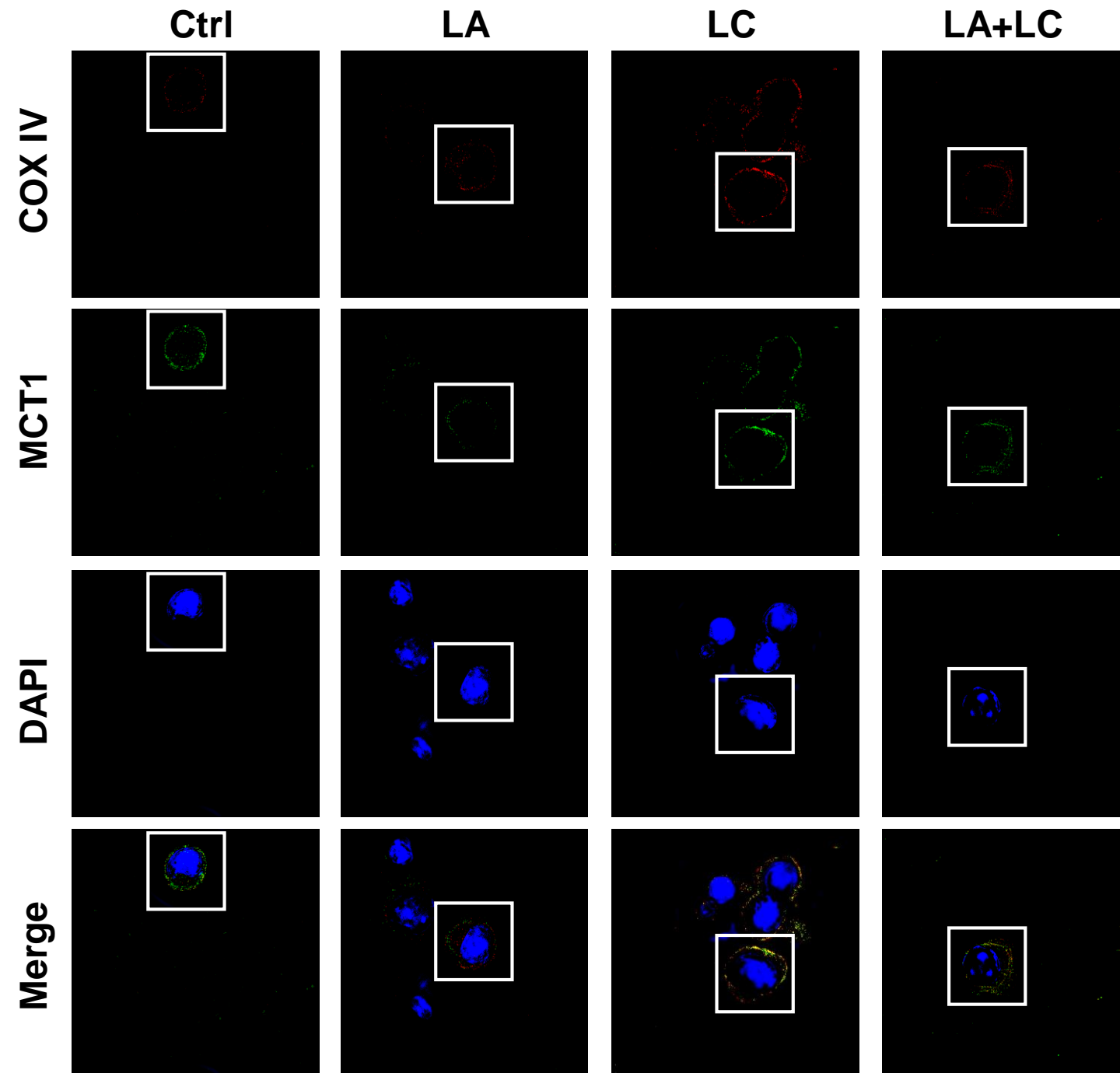

**Figure 5e**

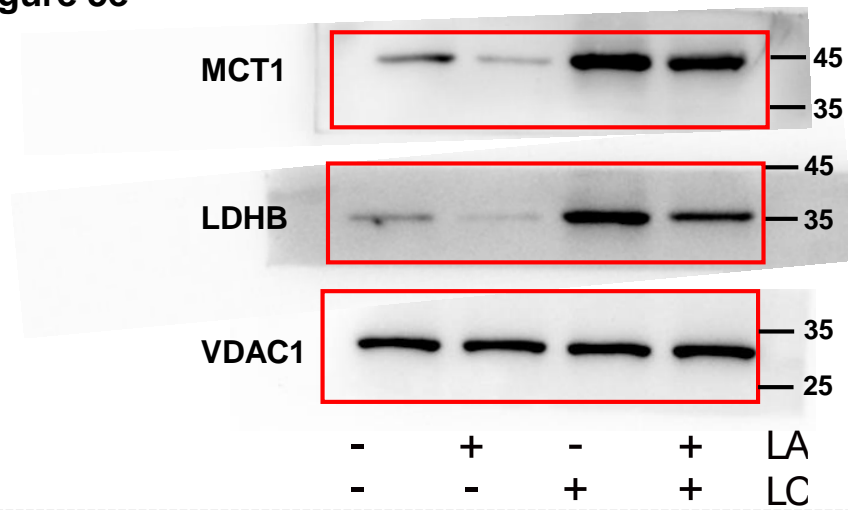

Supplement: Supplementary file 10 — Unprocessed fluorescence image and blots. [file 41590_2023_1738_MOESM10_ESM.pdf]

Extended Data Figure 1i

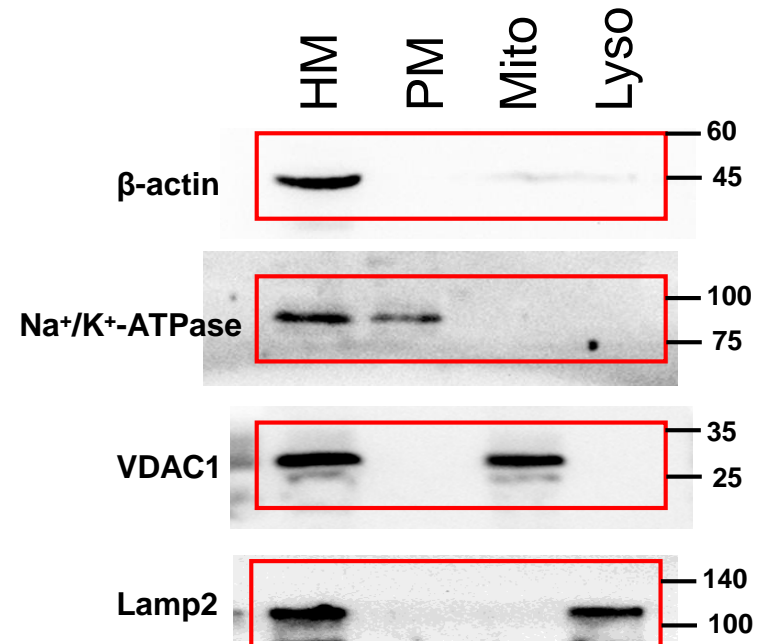

Supplement: Supplementary file 11 — Unprocessed blots [file 41590_2023_1738_MOESM11_ESM.pdf]

Extended Data Figure 4a

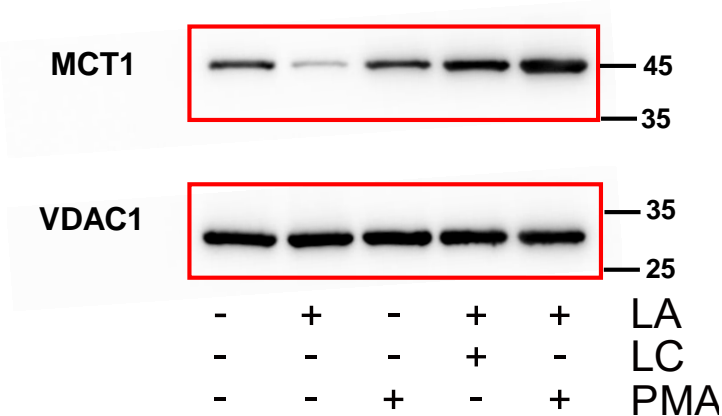

Extended Data Figure 4d

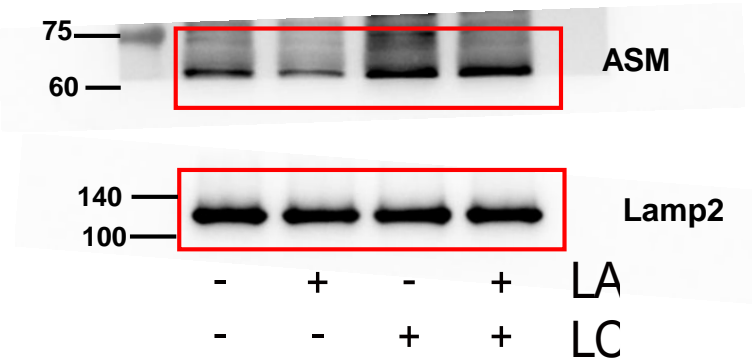

Extended Data Figure 4g

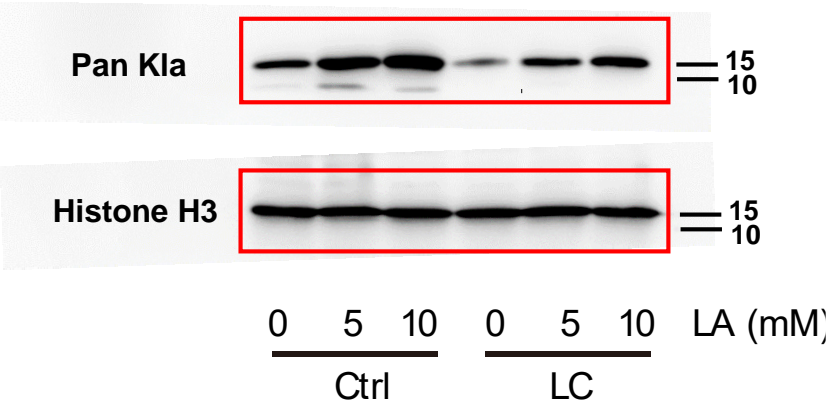

Extended Data Fig. 4c

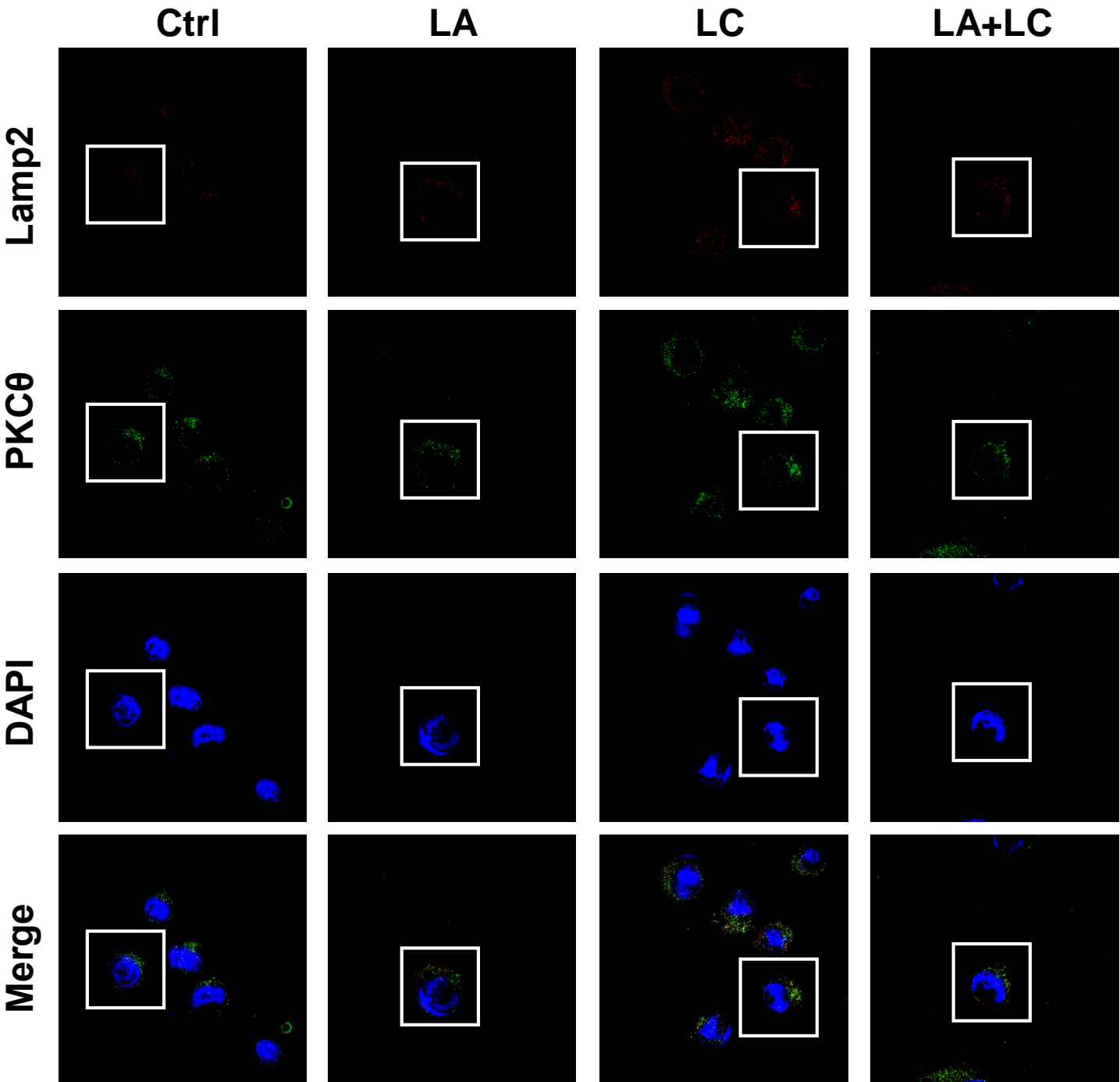

Supplement: Supplementary file 13 — Unprocessed fluorescence image and blots. [file 41590_2023_1738_MOESM13_ESM.pdf]

Extended Data Figure 5c

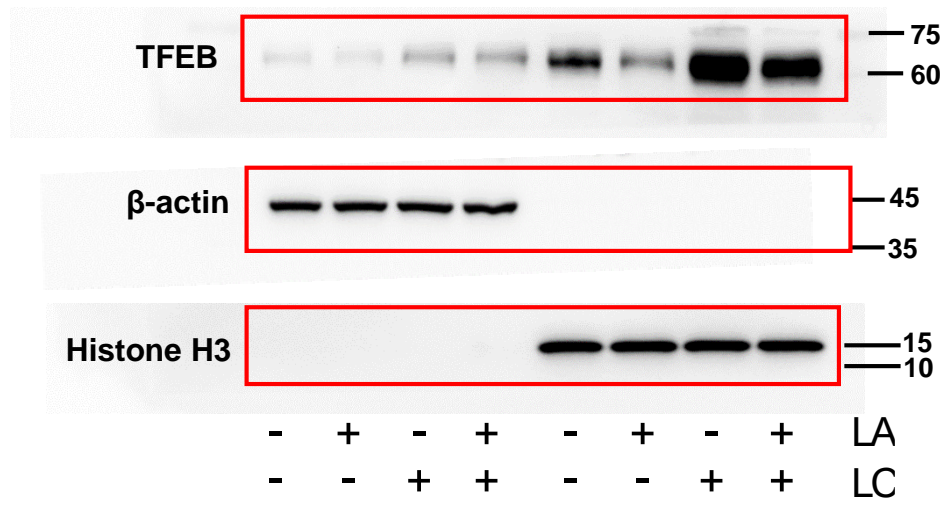

Extended Data Figure 5n

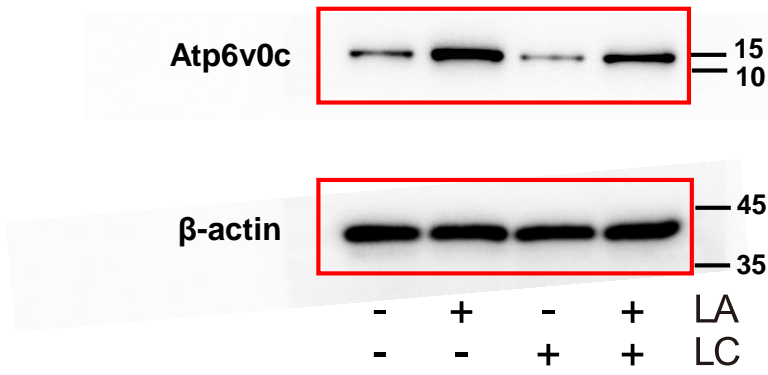

## Extended Data Fig. 5e

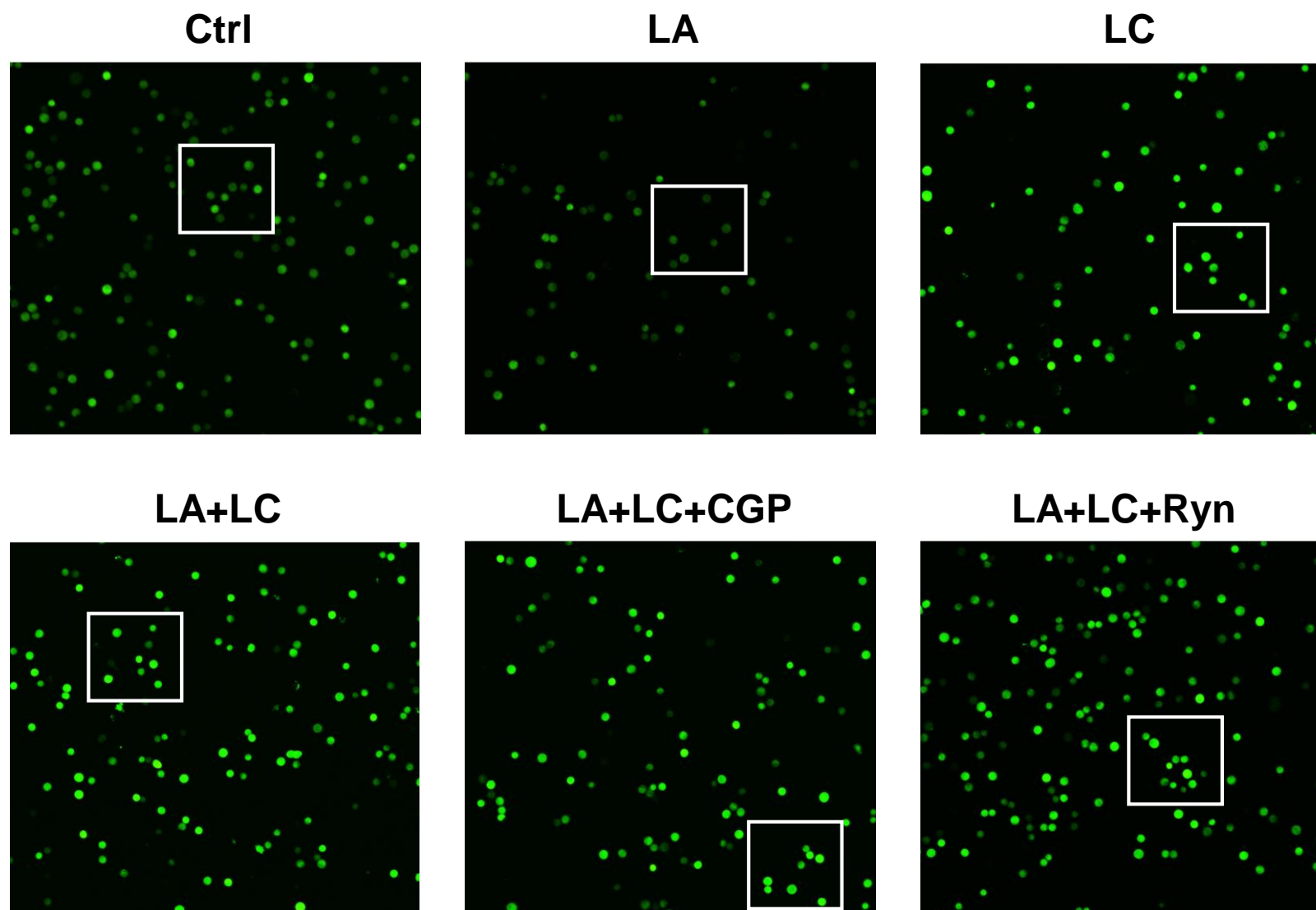

Supplement: Supplementary file 19 — Unprocessed fluorescence image and blots. [file 41590_2023_1738_MOESM19_ESM.pdf]

Extended Data Figure 7c

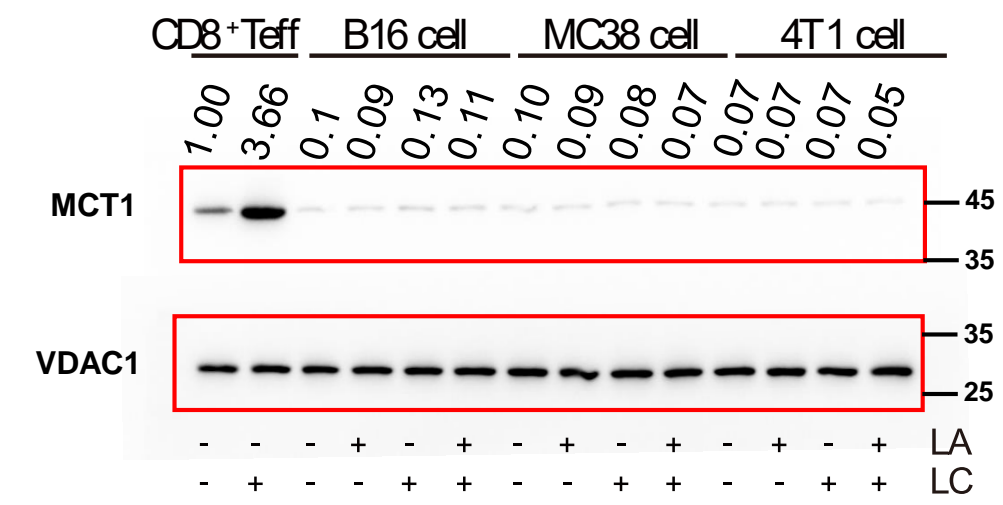

Extended Data Figure 7j

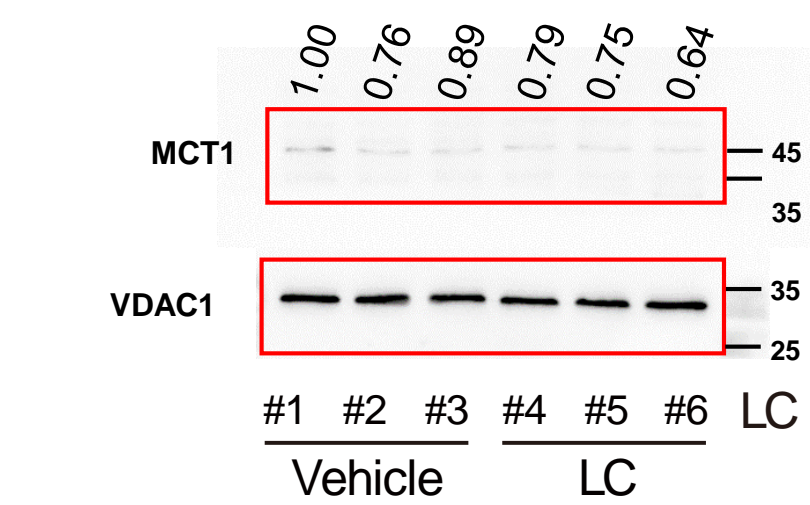

Supplement: Supplementary file 21 — Unprocessed blots [file 41590_2023_1738_MOESM21_ESM.pdf]

Extended Data Figure 9c

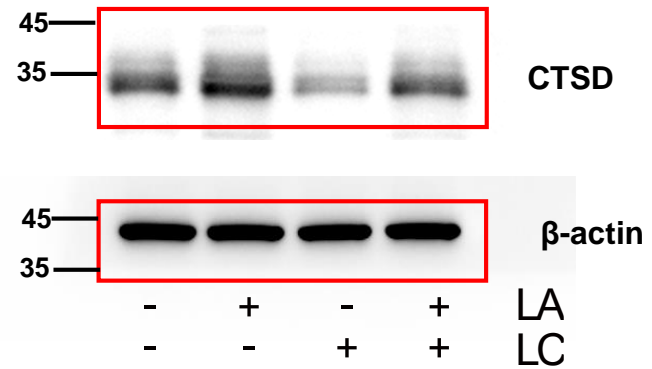

Extended Data Figure 9f

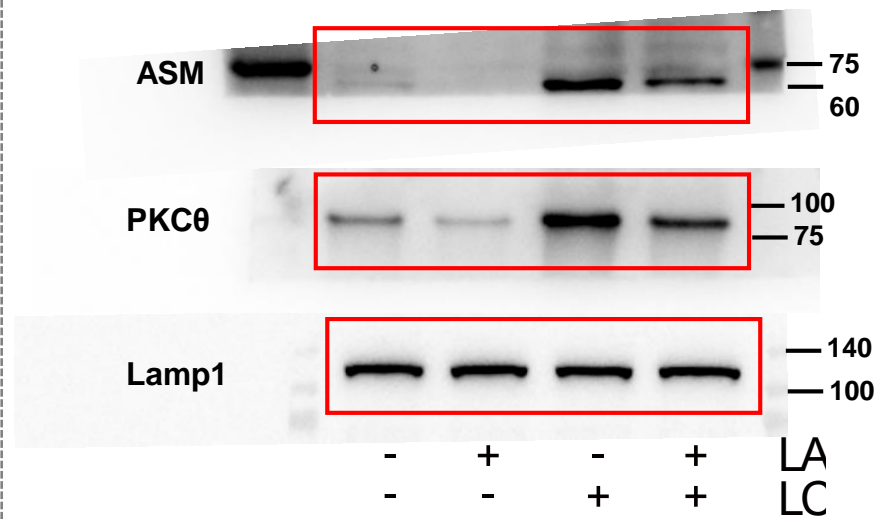

Extended Data Figure 9k

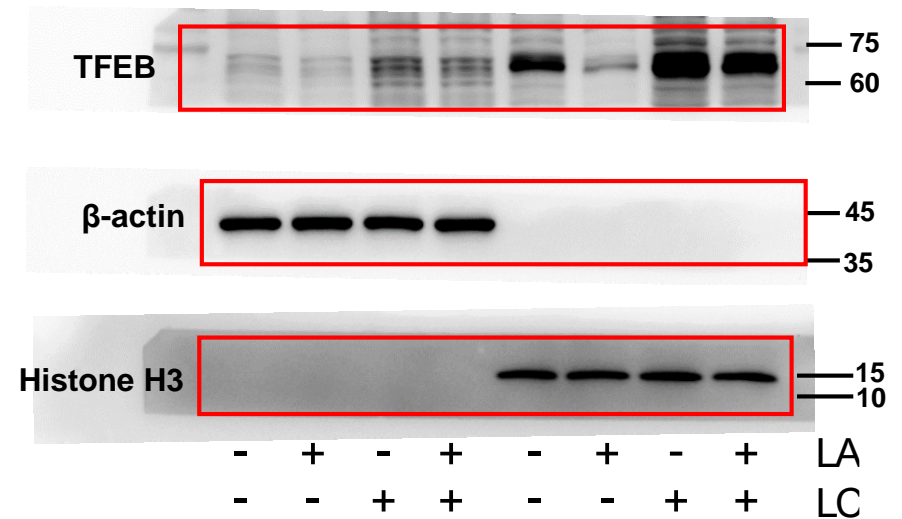

Extended Data Fig. 9g

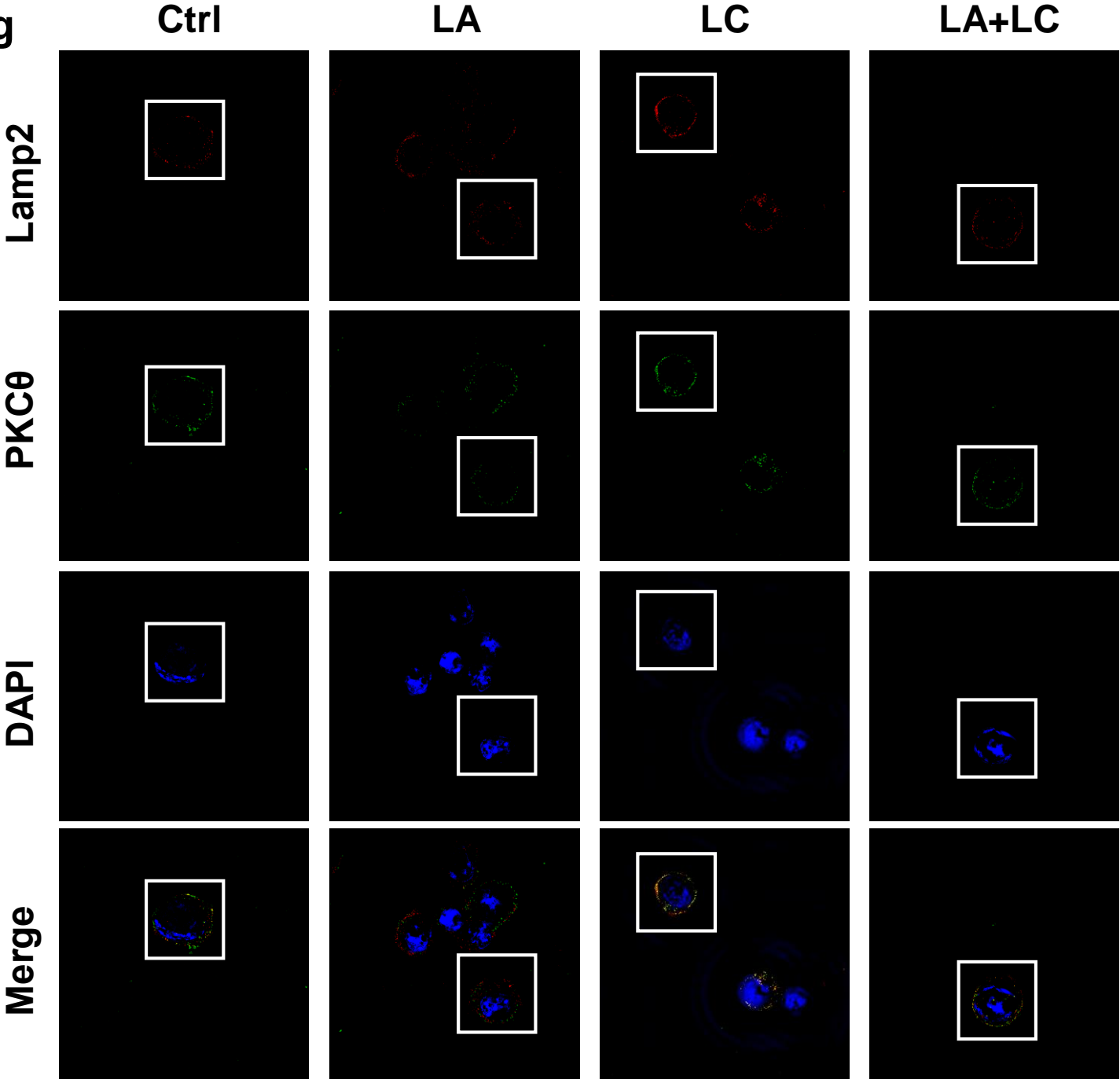

Supplement: Supplementary file 25 — Unprocessed fluorescence image and blots. [file 41590_2023_1738_MOESM25_ESM.pdf]
