# Supplementary material for: Lithium carbonate revitalizes tumor-reactive CD8+ T cells by shunting lactic acid into mitochondria
Source: Nat Immunol. 2024 Jan 23;25(3):552–61. doi: 10.1038/s41590-023-01738-0 (PMC10907288; doi:10.1038/s41590-023-01738-0)

Extended Data Fig. 3a

isotype MCT1

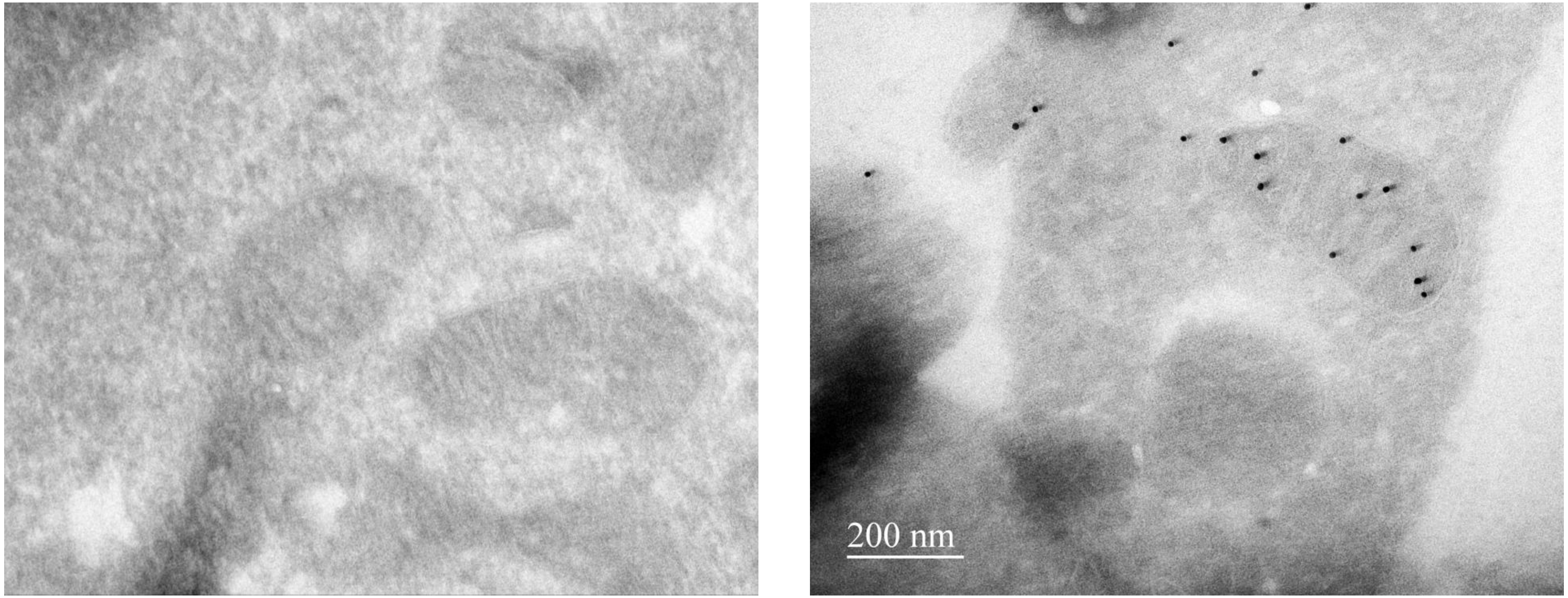

Extended Data Figure 3b

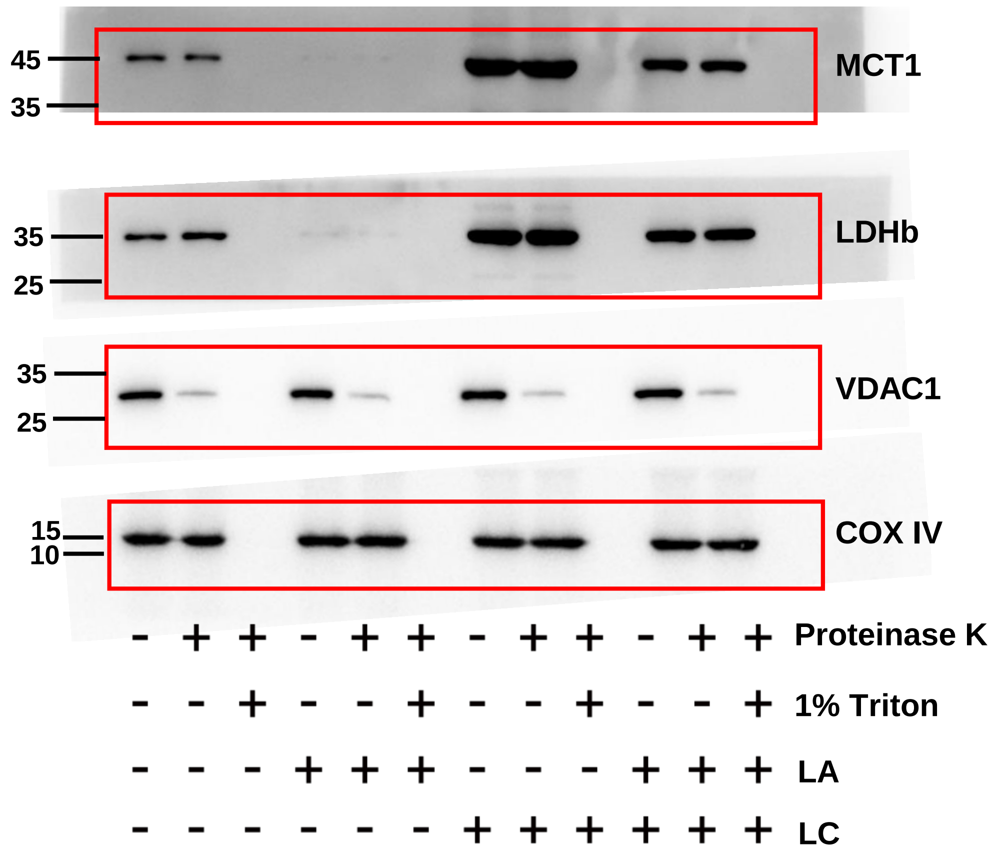

Extended Data Figure 3c

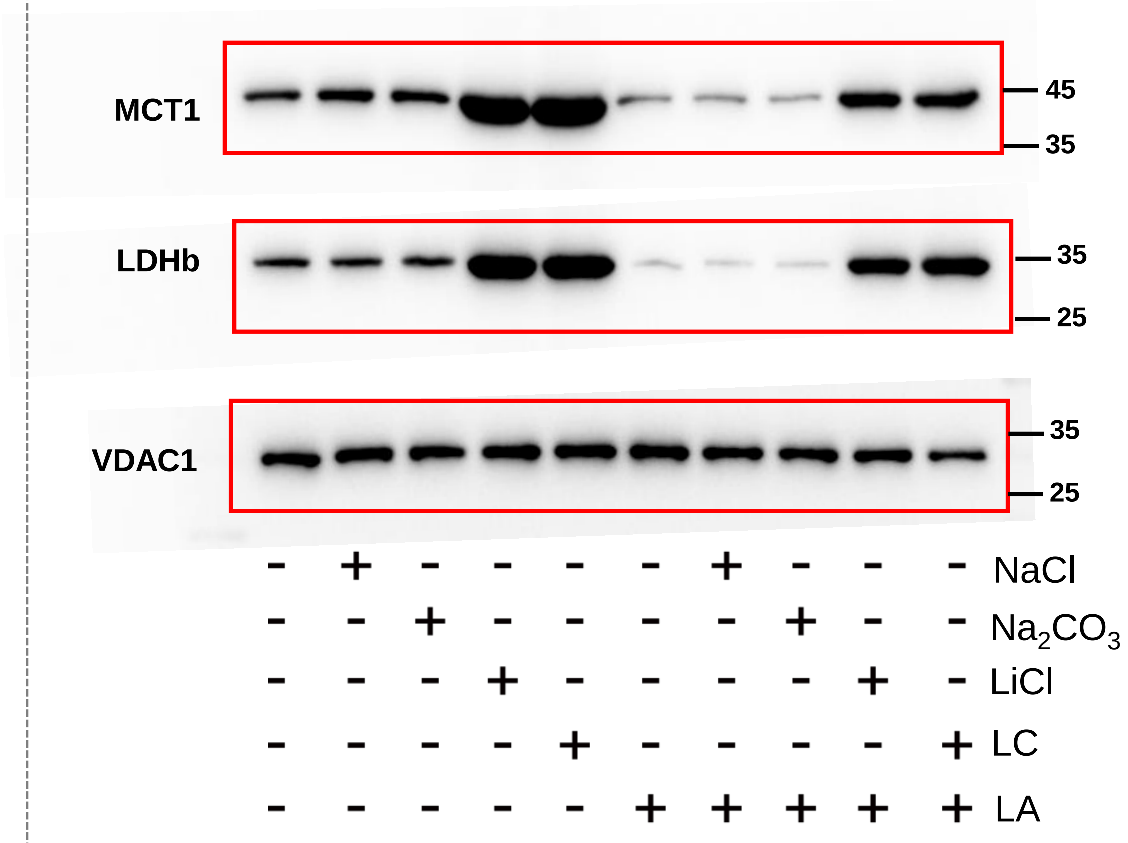

Extended Data Figure 3e

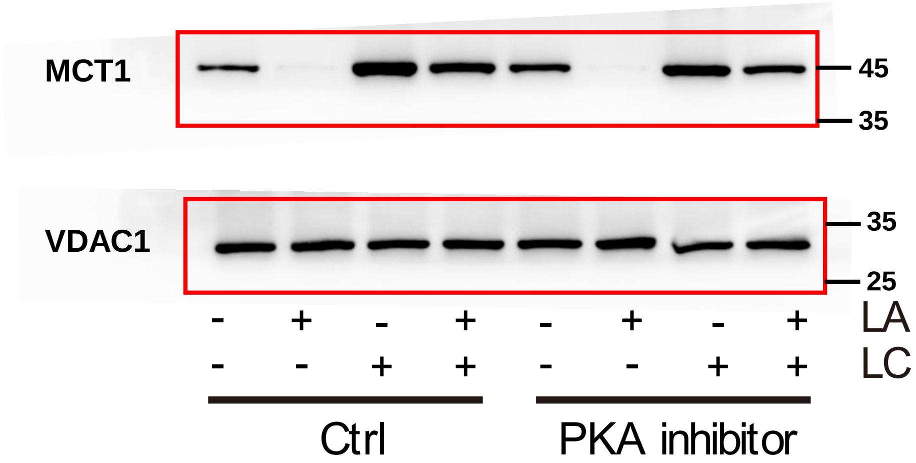

Extended Data Figure 3e

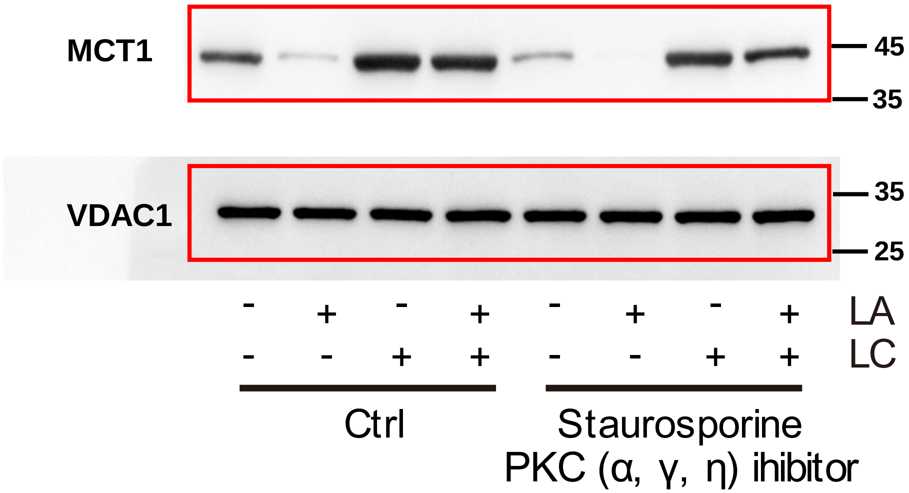

Extended Data Figure 3e

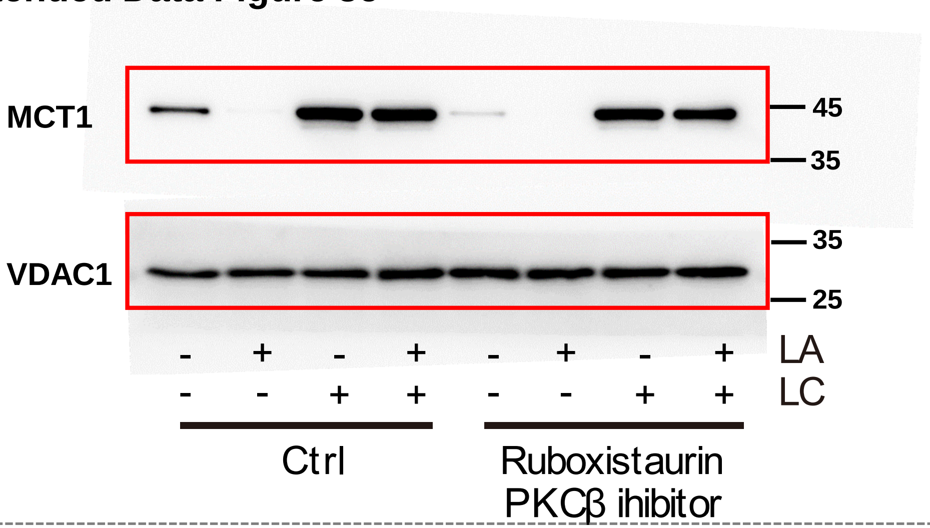

Extended Data Figure 3g

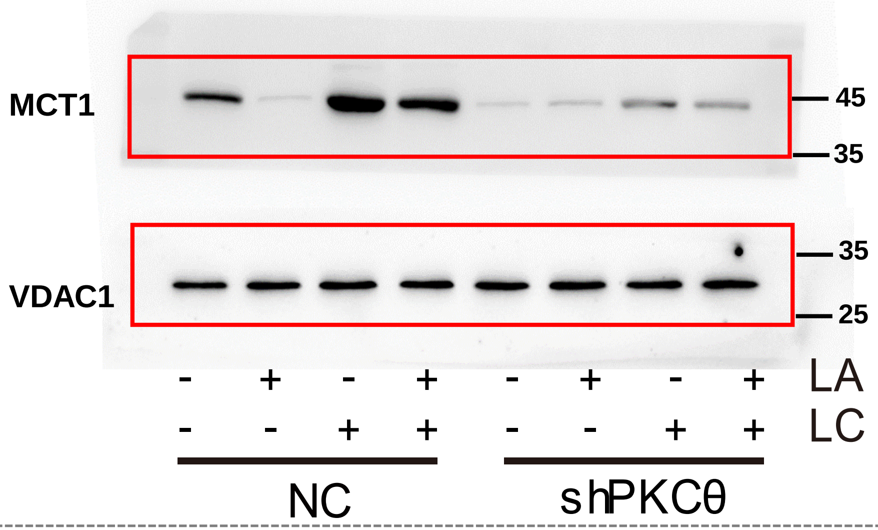

Extended Data Fig. 3i

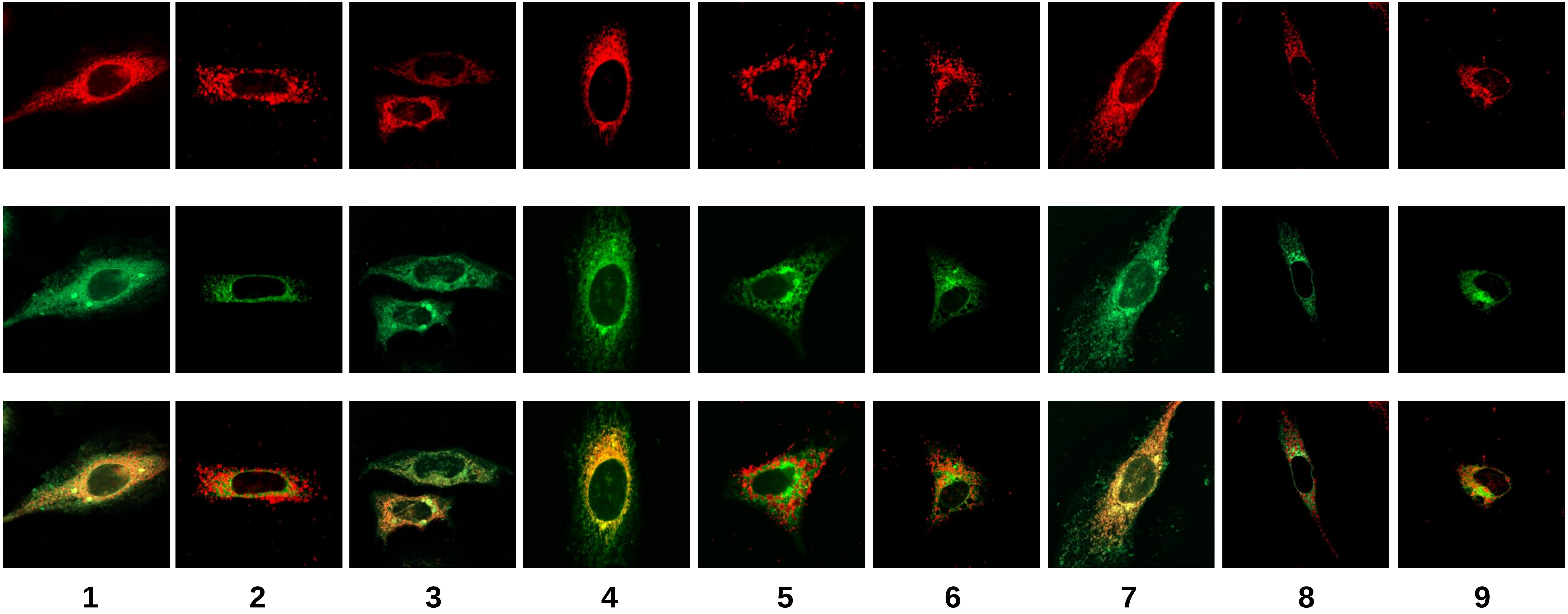

Supplement: Supplementary file 12 — Unprocessed fluorescence image and blots. [file 41590_2023_1738_MOESM12_ESM.pdf]
